# microRNA-22 Promotes Heart Failure through Coordinate Suppression of PPAR/ERR-Nuclear Hormone Receptor Transcription

**DOI:** 10.1371/journal.pone.0075882

**Published:** 2013-09-27

**Authors:** Priyatansh Gurha, Tiannan Wang, Ashley H. Larimore, Yassine Sassi, Cei Abreu-Goodger, Maricela O. Ramirez, Anilkumar K. Reddy, Stefan Engelhardt, George E. Taffet, Xander H. T. Wehrens, Mark L. Entman, Antony Rodriguez

**Affiliations:** 1 Department of Molecular and Human Genetics, Baylor College of Medicine, Houston, Texas, United States of America; 2 Department of Molecular Physiology and Biophysics, Baylor College of Medicine, Houston, Texas, United States of America; 3 Institute of Pharmacology and Toxicology, Technische Universitat Munchen, Munich, Germany; 4 Munich Heart Alliance, Munich, Germany; 5 Laboratorio Nacional de Genómica para la Biodiversidad, CINVESTAV, Irapuato, Guanajuato, México; 6 Department of Surgery, Baylor College of Medicine, Houston, Texas, United States of America; 7 Department of Medicine, Baylor College of Medicine, Houston, Texas, United States of America; Rutgers New Jersey Medical School, United States of America

## Abstract

Increasing evidence suggests that microRNAs are intimately involved in the pathophysiology of heart failure. *MicroRNA-22* (*miR-22*) is a muscle-enriched miRNA required for optimum cardiac gene transcription and adaptation to hemodynamic stress by pressure overload in mice. Recent evidence also suggests that *miR-22* induces hypertrophic growth and it is oftentimes upregulated in end stage heart failure. However the scope of mRNA targets and networks of *miR-22* in the heart failure remained unclear. We analyzed transgenic mice with enhanced levels of *miR-22* expression in adult cardiomyocytes to identify important pathophysiologic targets of *miR-22*. Our data shows that forced expression of *miR-22* induces a pro-hypertrophic gene expression program, and it elicits contractile dysfunction leading to cardiac dilation and heart failure. Increased expression of *miR-22* impairs the Ca^2+^ transient, Ca^2+^ loading into the sarcoplasmic reticulum plus it interferes with transcription of estrogen related receptor (ERR) and PPAR downstream genes. Mechanistically, *miR-22* postranscriptionally inhibits peroxisome proliferator-activated receptor gamma coactivator 1 alpha (PGC-1α), PPARα and sirtuin 1 (SIRT1) expression via a synergistic circuit, which may account for deleterious actions of unchecked *miR-22* expression on the heart.

## Introduction

Heart failure (HF) is a complex disorder defined by impaired myocardial contractility, deprivation of cardiac bioenergetics, increased myocyte cell death, and collagen scar formation. A major risk factor for HF comes from maladaptive cardiac hypertrophy, which occurs as a result of sustained hemodynamic overload due to chronic hypertension and/or cardiac injury [1,2].

At the cellular level pathological hypertrophy is characterized by an increase in cardiac myocyte cell size, increased protein synthesis, sarcomeric remodeling, and a switch to the hypertrophic molecular gene program that mimics that observed in fetal heart development [3]. Pathologic cardiac hypertrophy is controlled at multiple molecular levels including mechanosensation, cytoplasmic signal transduction, transcriptional, and postranscriptional mechanisms. Large numbers of transcription factors and coregulators such as nuclear factor of activated T-cells (NFAT), GATA4, sirtuin 1 (SIRT1), estrogen related receptor (ERR-α,β,γ), peroxisome proliferator-activated receptor (PPAR-α,β,γ), and peroxisome proliferator-activated receptor gamma coactivator 1 alpha (PGC-1α) contribute to pathological hypertrophy traits [2,4,5]. PGC-1α, PPARα, and ERRα are frequently downregulated in hypertrophic myocytes and diseased hearts [5,6]. PGC-1α interacts and co-activates the ERR/PPAR nuclear hormone receptor superfamilies as well as other non-nuclear hormone transcription factors to meet contractile and bioenergetic demands of the heart during stress [7]. PPARα, the main cardiac PPAR member, activates the transcription of genes stimulating fatty acid transport and oxidation; and, downregulation of PPARα is thought to constitute a nodal pathogenic switch responsible for decreased capacity for fatty acid utilization toward glucose catabolism in stressed hearts [6,8]. ERRα is a key regulator of ATP synthetic capacity and oxygen consumption in the heart required for mitochondrial fatty acid and glucose oxidation, Krebs cycle activity, oxidative phosphorylation (OXPHOS), and calcium handling protein expression [9,10]. The regulatory events driving coordinate downregulation of PPARα, PGC-1α, and ERRα expression/activity in stressed hearts remain poorly understood, but it is thought they contribute to definitive characteristics of maladaptive remodeling and HF [5–8].

A large body of evidence indicates that microRNA (miRNA)-mediated gene regulation represents an important layer of control of cardiac hypertrophy and heart failure [11]. Independent *in vivo* studies in mice suggest that the heart enriched, *microRNA-22* (*miR-22*) is involved in the pathogenesis of HF [12,13]. We previously showed that *miR-22*-deficient mice exhibit increased fibrosis and increased tendency towards ventricular dilatation and cardiac dysfunction in response to pressure overload [12]. In this study, we set out to decipher the molecular and pathological consequences of enforced *miR-22* expression on the heart. Extending previous observations, we show that cardiac-specific enforced expression of *miR-22* in mice elicits contractile dysfunction and HF. Using a combination of gain-of-function (GOF) experiments in mice and cultured cardiomyocytes, we demonstrate that *miR-22* directly inhibits PGC-1α, PPARα, and SIRT1 expression levels leading to HF.

## Materials and Methods

### Animal Models

The generation of the two *miR-22* transgenic cardiomyocyte-specific mice lines (herein referred to as TG-M and TG-H) [12], in which *miR-22* was placed under the control of the *Myh6* promoter, were previously described. The TG-M and TG-H mice were maintained in an FBV isogenic background. The mice harboring the null *miR-22* mutant allele was described earlier [12]. All animal procedures were approved by the Baylor College of Medicine Institutional Animal Care and Use Committee (Animal Protocol 4930).

Mice were anesthesized with 1.5% isoflurance by inhalation. Echocardiography was performed in M mode with a Vevo 770 RMV-707B (Visual Sonics) instrument as described earlier [12]. Dobutamine was injected at 3µg/g by intraperitoneal injection and hemodynamic measurements were made with a Millar catheter (SPR1000, Millar Instruments, Houston, TX). Doppler flow measurements and electrocardiograms made use of a 10 MHz Doppler probe connected to a Doppler Signal Processing Workstation (Model DSPW, Indus Instruments, and Houston, TX, USA) to measure aortic outflow and mitral inflow velocities from the cardiac apex. After an optimal signal was achieved, two-second segments were acquired and analyzed.

### Histological analysis and cell size measurement

Histological analysis of hypertrophy, fibrosis, and apoptosis was performed on hearts fixed overnight in formalin, dehydrated, and processed into paraffin blocks for sectioning. Serial 5 µm sections were cut and stained by Masson trichrome, or picrosirius red. For cell surface area measurements, heart sections were stained with TRITC-labeled lectin (catalog L5266, Sigma) as previously described [12]. Terminal deoxynucleotidyl transferase-mediated dUTP nick end labeling (TUNEL) was measured using the TMR Red *in situ* detection kit according to the manufacturer’s instructions (Roche Diagnostics). Image-Pro Plus imaging (Media Cybernetics) software was used for histomorphometric quantification.

### Quantitative PCR analysis

Total RNA was extracted from mice hearts or NRVC as previously described [12,14]. Expression assays in mice made use of the High Capacity cDNA Reverse Transcription Kit and Taqman Universal Master mix for cDNA preparation and quantitative PCR respectively. Expression assays in mice made use of pre-designed Taqman probes (Life Technologies). Custom-designed SYBR green probes were used for expression assays in NRVC. Rat probes were designed utilizing the Integrated DNA Technologies real time PCR tool (http://www.idtdna.com/Scitools/Applications/RealTimePCR/). *Gapdh* was used as a normalization control. SYBR probes were quality controlled by analyzing the corresponding dissociation curve for each amplicon and by ensuring sequence uniqueness in genome BLAST search.

### Cell culture, miRNA transfection, luciferase reporter assays

Primary cultures of rat neonatal cardiomyocytes (NRVC) were established from 1-2 day old Sprague Dawley rats as previously described [14]. Cardiomyocyte cell size determination, phenylephrine treatment, and immunohistochemistry staining were as described previously [14]. Transfection of miRNA mimics was achieved following recommended guidelines for Lipofectamine 2000. Cardiomyocyte lysates were collected 48 hrs after transfection [14].

Target gene 3’ UTRs were PCR amplified utilizing murine genomic DNA and cloned into the psiCHECK2 luciferase reporter plasmid (Promega). The miRNA luciferase reporter assays were carried out in 3T3 mouse embryonic fibroblasts (MEFs) as described earlier [12]. Transfections made use of Oligofectamine (Invitrogen) with 100 ng of indicated psiCheck-2 plasmid containing wild-type or *miR-22* ‘seed’ mutant derivatives, along with the miRNA control or *miR-22* duplex (Dharmacon) at a final concentration of 6 nM. Lysates were collected 24 hrs after transfection and luciferase activity measured by Dual-Luciferase Reporter System (Promega).

### Microarray and gene set enrichment analysis

Transcriptome microarrays were carried out utilizing the Illumina Mouse WG-6 v2 Whole-Genome Expression Beadchips as described earlier [12]. FDR was estimated using the Benjamini and Hochberg method and probes were considered to be significantly differentially expressed if the adjusted p-value was < 0.05 (5% FDR) and the fold-change was > 1.2. Sylamer and gene set enrichment analysis (GSEA) analyses were applied as previously described [12]. The microarray data was deposited into ArrayExpress as E-MTAB-501.

### Immunoblotting

Western blots were performed on cardiac whole cell lysates or cardiomyocyte lysates prepared with RIPA extraction buffer. Primary antibodies used were: CAV3 (1:1500, catalog 610420; BD Biosciences), PGC-1α (1:2000, Catalog ab54481; Abcam), PPARα (1:1000, catalog ab8934; Abcam), SIRT1 (1:2000, catalog 07-131; Upstate Biotechnology) and GAPDH (1:5000, catalog AM4300; Ambion). Secondary antibodies used were: goat anti-rabbit IgG (1:10,000, catalog A21077, Invitrogen) and donkey anti-mouse IgG (1:10000, catalog 926-32212; LI-COR Biosciences). GAPDH was detected as a loading control. Quantitative densitometry was used to calculate protein levels using an Odyssey infrared imaging system (LI-COR Biosciences). Digital images of bands of interest were processed for better contrast and cropped using Photoshop software (Adobe Systems).

### Adult myocyte isolation and calcium handling assays

Adult ventricular myocytes were isolated as described previously [12]. Briefly, the heart was cannulated through aorta and firstly perfused with 0 Ca^2+^ Tyrode solution (137 mM NaCl, 5.4 mM KCl, 1 mM MgCl_2_, 5 mM HEPES, 10 mM glucose, 3 mM NaOH, pH 7.4) on a langendroff system. After washing for 5 minutes at 37°C by 0 Ca^2+^ Tyrode, the heart was digested by 0.5mg/ml collagenase for 25min. The myocytes were mechanically separated in KB solution (90 mM KCl, 30 mM K_2_HPO_4_, 5 mM MgSO_4_, 5 mM pyruvic acid, 5 mM β-hydroxybutyric acid, 5 mM creatine, 20 mM taurine, 10 mM glucose, 0.5 mM EGTA, 5 mM HEPES, pH 7.2) and gently agitated, then filtered through a 210 mm polyethylene mesh. For calcium imaging, rod-shaped ventricular myocytes with clear striation were loaded with 2 µM Fluo-4/AM (Invitrogen, Carlsbad, CA) in normal Tyrode solution containing 1.8 mM Ca^2+^ for 30 min at room temperature. Then, cells were transferred to a chamber equipped with parallel platinum electrodes and placed on a LSM510 confocal microscope (Carl Zeiss, Thornwood, NY).

Steady-state Ca^2+^ transients were triggered by 1Hz-pacing (10ms interval, 15V) protocol as previously described [15]. When pacing was stopped, perfusate was switched to a 0 Na^+^ /0 Ca^2+^ Tyrode solution which blocks the Ca^2+^ exchange via the Na/Ca exchanger and the L-type Ca^2+^ channel. Acute application of 10mM caffeine under 0 Na^+^ /0 Ca^2+^ was used to estimate SR load [12]. The activity of SERCA was estimated by the transient decay under 1Hz-pacing, and the activity of NCX was estimated as the caffeine-triggered transient decay under 0 Na^+^ /0 Ca^2+^ Tyrode solution as previously described [15]. Data from at least ten cells from each mouse heart were used for analysis.

### Statistical Analyses

Quantitative data are expressed as the means +/- SEM. Statistical differences between experimental groups were determined using the two-tailed Student’s *t* test, 1 or 2-way ANOVA with fisher LSD, Dunnett or Tukey post-hoc test. The Mann-Whitney U test was used to compare continuous variables with a skewed distribution. *P* < 0.05 was considered statistically significant.

## Results

### Cardiomyocyte-specific overexpression of *miR-22* promotes hypertrophic growth and cardiomyopathy

**Figure 1:**
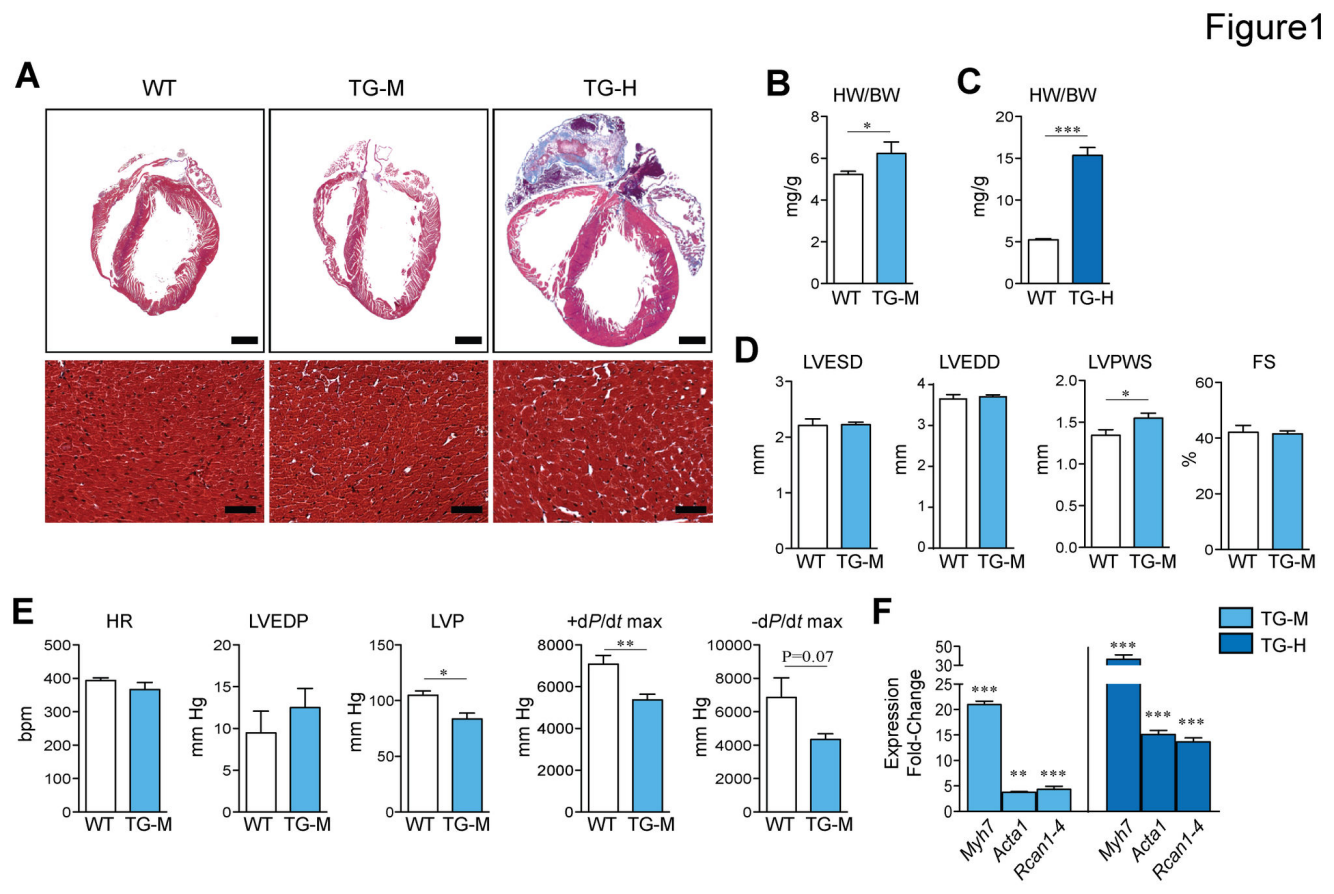
Enforced expression of *miR-22* in the heart is sufficient to induce cardiomyopathy. (A) Representative hearts of TG-M, TG-H and non-transgenic (WT) mice at 12-weeks of age. Top and bottom panels show Masson’s Trichrome stained sections at low and high magnification respectively (Scale bars: 1mm and 50mm). (B,C) Heart weight normalized to body weight (HW/BW) in 12-week old TG-M and TG-H mice versus WT (*n* = 5-12). (D) Analyses of cardiac function by echocardiography in 12-week old TG-M and WT mice. LVESD, LV end-systolic dimension; LVEDD, LV end-diastolic dimension; LVPWS, LV posterior wall thickness at end-systole; FS, fractional shortening (*n* = 10-11). (E) Hemodynamic analysis with a Millar catheter in 12-week old TG-M and WT mice. HR, heart rate; LVEDP, LV end-diastolic pressure; LVP, maximal LV pressure; +dP/dt max, maximal contraction rate; -dP/dt max, maximal relaxation rate (*n* = 5). (F) Relative mRNA expression levels of indicated genes in 12-week old TG-M and WT mice. Bars represent expression normalized with WT set equal to 1.0 (*n* = 3-4). Student *t* test. *, P<0.05; **, P<0.01; ***, P<0.001.

We previously described cardiac myocyte-specific *miR-22* overexpressing transgenic mice that showed signs of cellular and organ level hypertrophic growth compared to their WT littermates beginning at 5-weeks of age [12]. The level of *miR-22* expression in the TG-M and TG-H mice were 4- and 9-fold higher respectively in comparison to non-transgenic littermate (WT) mice [12]. As expected, gravimetric measurements of cardiac mass showed significantly increased hypertrophy in 12-week old TG-M and TG-H mice in comparison to WT mice (Figure 1B, 1C). Initially we focused on the functional phenotype of the lower-expressing transgenic line. Baseline cardiac structure and function of TG-M mice was similar to WT mice at 5-weeks of age (Table 1 and data not shown), but by 12-weeks of age TG-M mice showed a basal pattern of hypertrophy as evidenced by left ventricular (LV) systolic posterior wall thickness (LVPWS) compared to WT mice (Figure 1D). No changes in fractional shortening (FS) were evident in mice of this age (Figure 1D). However, closed chest hemodynamic analysis detected a 20% reduction in maximal LV pressure; furthermore, LV maximal contraction (dP/dt_max_) and LV maximal relaxation rate (-dP/dt_max_) were repressed by 24% and 37% suggesting that cardiac contractility was reduced in TG-M mice (Figure 1E).

**Table 1:** Pulsed Doppler echocardiographic analysis of 5-week old TG-M, TG-H mice and WT controls.

Parameter	WT		TG-M		TG-H
*n*	4		5		8
HR (bpm)	323 ± 10		351 ± 18		243 ± 12***
LA vol (mm^3^).	4.17 ± 0.33		nd		6.98 ± 0.98*
LVPWS (mm)	1.23 ± 0.05		1.18 ± 0.08		1.49 ± 0.11
LVPWD (mm)	1.02 ± 0.05		0.78 ± 0.08		1.05 ± 0.08
LVESD (mm)	2.01 ± 0.11		2.32 ± 0.08		1.99 ± 0.14
LVEDD (mm)	3.50 ± 0.02		3.78 ± 0.09		3.87 ± 0.09*
FS	42.7 ± 3.03		40.5 ± 1.41		48.6 ± 3.10
IVRT (msec)	16.00 ± 1.0		17.30 ± 1.5		20.3 ± 1.0
PAoFV (cm/sec)	108.0 ± 4		120.3 ± 4		119.0 ± 6
PEV (cm/sec)	78 ± 3		81 ± 1		83 ± 4

HR, heart rate; LA vol, Left Atrial volume; LVPWS, Left Ventricular(LV) end-systolic posterior wall thickness; LVPWS, Left Ventricular(LV) end-diastolic posterior wall thickness; LVESD, LV end-systolic dimension; LVEDD, LV end-diastolic dimension; FS, fractional shortening; IVRT, Isovolumic relaxation time; PAoFV, Peak Aortic Flow Velocity; PEV, Peak Ejection Flow VelocityData were analyzed by 1 way ANOVA. *, P<0.05; *** P<0.005 versus WT.

To gain more insight into the pathologic effects associated with enforced *miR-22* cardiac expression, echocardiographic and Doppler flow measurements and electrocardiograms were obtained from the higher *miR-22* expressing TG-H line. Echocardiography analysis revealed atrial and ventricular chamber growth, as indicated by significant increases in LV end diastolic dimension (LVEDD) and left atrial volume (LA Vol) in 5- and 8-week old TG-H mice (Table 1 and Table S1). Echocardiography showed that the cell and organ level hypertrophy in these mice were not associated with thickening of the free wall despite the dramatic increase in myocyte cross sectional area [12]. Average heart rate was dramatically lower beginning at 5-weeks of age in TG-H mice compared with WT mice (Table 1 and Table S1). Furthermore, Doppler and electrocardiographic recordings showed increased coefficient of variation in R-R intervals (CVR-R) and wide variations in stroke volume evocative of atrial fibrillation in TG-H mice (Table S1 and Figure S1). By 8-weeks of age TG-H mice showed signs of impaired diastolic function as indicated by the profound elongation in isovolumetric relaxation time (IVRT) (Table S1).

To further investigate potential differences in *miR-22* transgenic mice versus WT controls, hearts were subjected to histological and molecular analysis. Histological appearance of ventricular myocardium of TG-M and TG-H mice was similar to WT mice up to 12-weeks of age (Figure 1A). Specifically, fibrosis or cellular disarray was not a prominent feature as indicated by examination of paraffin embedded heart sections stained with Masson trichrome or picrosirius red. However terminal deoxynucleotidyl transferase dUTP nick end- labeling was greater in TG-H than in control hearts indicative of enhanced myocardial apoptosis (Figure S2). Furthermore, TG-H mice had large atrial clots, consistent with poor cardiac function (Figure 1A). Quantitative polymerase chain reaction (qPCR) analysis revealed elevated mRNA levels of hypertrophic markers [e.g. sarcomeric actin alpha 1 (*Acta1*) and β-cardiac muscle myosin heavy chain (*Myh7*)] and NFAT activation [i.e. regulator of calcineurin 1 splice variant 4 (*Rcan1-4*)] in ventricles of TG-M and TG-H mice between 5- and 12-weeks of age (Figures 1F and [12]). Taken as a whole, these data indicate that enforced *miR-22* expression in heart is deleterious.

### *miR-22* inhibits a large number or genes associated with cell growth, cell death, and energy substrate metabolism

As an initial step to delineate *miR-22* mRNA targets and downstream networks, we performed genome wide gene expression analysis via microarray on ventricles of 12-week old TG-H and WT controls. The microarray identified many up and down-regulated genes in TG-H hearts (Table S2). To explore direct *miR-22*-mediated miRNA-mRNA interactions, we applied the Sylamer tool on the microarray [12]. Sylamer showed prominent miR-mediated mRNA destabilization since the motifs complementary to the common “seed” region of *miR-22* were specifically enriched within the 3’ UTRs of downregulated genes (Figure S3A). A comprehensive annotation and search of *miR-22* motifs revealed 276 repressed genes with *miR-22* seed matches in their 3’ UTR (Figure S3B and Table S2). We combined this list with the potential targets we identified earlier in *miR-22* ^*-/-*^ hearts [12]. This led to a total of 349 transcripts of which 284 were annotated by Ingenuity (Table S3). Ingenuity analysis revealed many dozens of *miR-22* target candidates associated with “cellular growth and proliferation”, “lipid metabolism”, and “cardiovascular disease” respectively (Table S3). A subset of genes containing highly conserved *miR-22* motifs in the 3’ UTR and linked to cardiomyopathy, including *calmodulin binding transcription* activator *2* (*Camta2*), *Caveolin-3* (*Cav3*), *Homer-1*, and *Pgc1a* were confirmed as repressed in *miR-22* transgenic hearts by quantitative PCR and/or immunoblot (Figure S3C-F and see below).

### *miR-22* regulates PGC-1α, PPARα and SIRT1 cardiac expression levels

**Figure 2:**
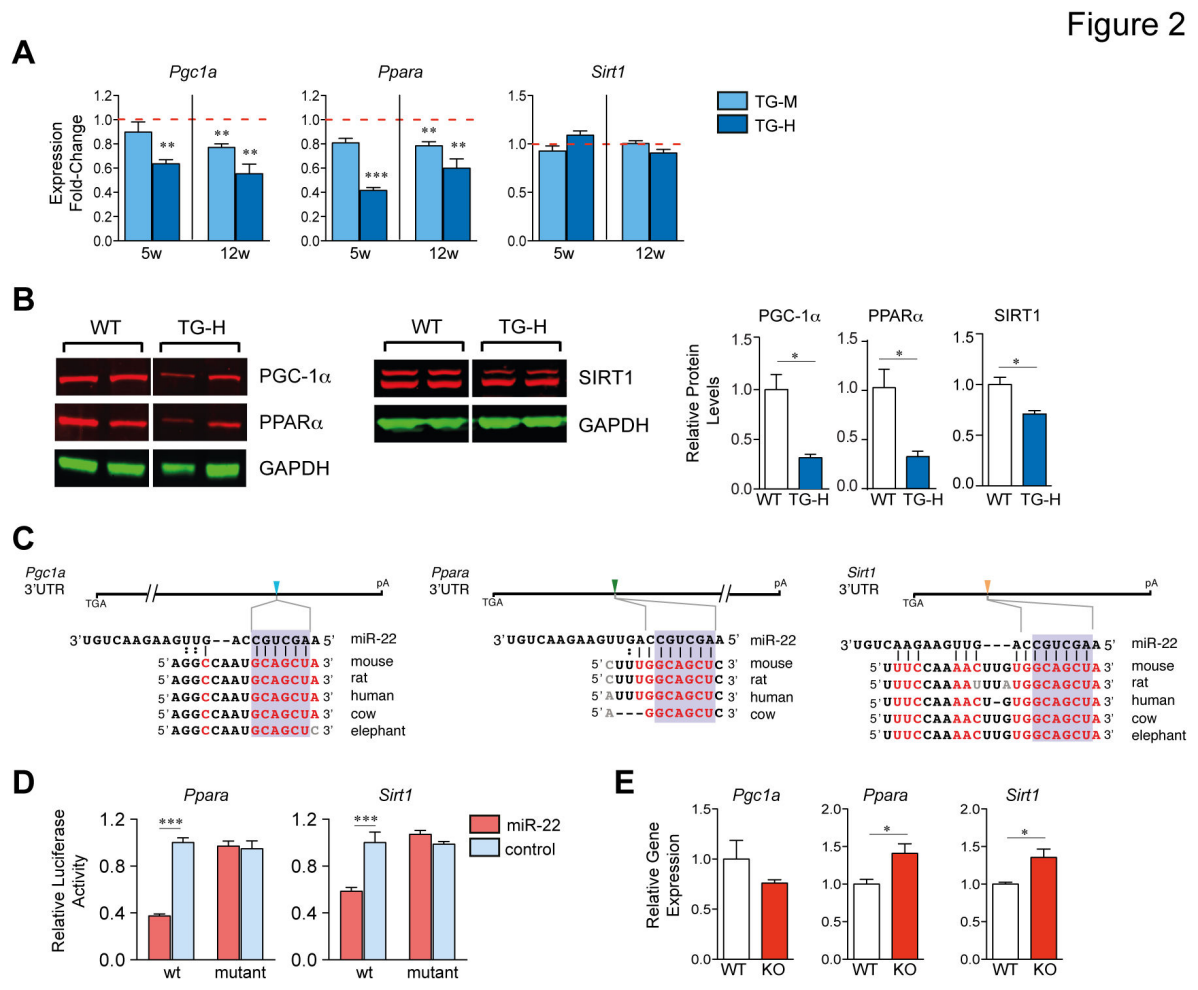
miR-22 co-represses PGC-1α, PPARα, and SIRT1 in the heart. (A) *Pgc1a*, *Ppara* and *Sirt1* mRNA was quantified by real-time PCR from ventricles of 5- (5w) or 12-week (12w) old mice of indicated genotypes. Bars represent expression normalized with WT set equal to 1.0 (*n* = 3-4). (B) Representative Western blot and quantitation of PGC-1α, PPARα, and SIRT1 levels from ventricular lysates obtained from 12-week old TG-H and WT mice. GAPDH was used as a loading control (*n* = 3 immunoblots from three mice each genotype). (C) *Pgc1a*, *Ppara* and *Sirt1* each contain highly conserved *miR-22*-mRNA interaction motifs within their 3’ UTRs. (D) Luciferase activity in 3T3 MEF cells transfected with indicated wt or site mutant (mut) 3’ UTR reporter constructs in the presence of *miR-22* or control miR mimic. Data are from two experiments carried out in triplicate. (E) Cardiac *Pgc1a*, *Ppara* and *Sirt1* mRNA expression levels were evaluated by qPCR from 6-week old *miR-22*-deficient (KO) and WT mice (*n* = 3). Student *t* test, (A,B,E); or 2-way ANOVA with the Tukey post hoc test, (D). *, P<0.05; **, P<0.01;.***, P<0.001.

A large number of important transcription factors or regulators implicated in cardiomyopathy have been identified as bona-fide targets of *miR-22*, including *Purb* [12], *Hdac4* [13], *Ppara* [16], *Sirt1* [17], and *Pgc1a* [18]. Loss-of-function genetic mutations in PGC-1α or PPARα in animal models are associated with HF [7–9,19–21]. To explore if *Pgc1a*, *Ppara* and *Sirt1* operate downstream of *miR-22* in heart, we examined 3’ UTRs of these three genes for *miR-22* binding motifs. As shown in Figure 2C, *Pgc1a*, *Ppara* and *Sirt1* each contain highly conserved complementary sequences to *miR-22*. We cloned the 3’ UTRs of *Ppara* and *Sirt1* into luciferase constructs. Reporter assays with *miR-22* expressing cells independently confirmed that *miR-22* represses these genes (Figure 2D). Mutation of the putative *miR-22* sites abrogated repression by *miR-22*, thus confirming functionality of sites (Figure 2D).

To determine if enforced expression of *miR-22* suppresses PGC-1α, PPARα and SIRT1 expression levels in concert, initially we performed qPCR and immunoblots on *miR-22* transgenic and WT mice. *Pgc1a* mRNA and protein levels were repressed by 1.8- and 3.2-fold respectively in 12-week old TG-H mice as compared to WT mice (Figure 2A, 2B). Furthermore, *Ppara* transcript and protein levels were reduced by 1.6- and 3.7-fold respectively in TG-H mice (Figure 2A, 2B). Protein levels of SIRT1 showed a 1.4-fold decrease in the absence of overt changes in mRNA evocative of a post-transcriptional negative effect (Figure 2A, 2B and data not shown). Consistent with expression analysis in TG-H mice, *Pgc1a* and *Ppara* mRNA levels were also reduced in 5- and 12-week old TG-M mice versus WT littermates (Figure 2A). We then asked whether *in vivo* absence of *miR-22* [12] in the heart would lead to coordinate accumulation of *Pgc1a, Ppara, Sirt1* transcript levels. Transcript levels for *Sirt1* and *Ppara* were each increased but *Pgc1a* appeared unchanged in 6-week old *miR-22*-null homozygous hearts compared to WT (Figure 2E).

To minimize the concern that changes in PGC-1α and PPARα expression levels are secondary to cardiac dysfunction and/or hypertrophy in transgenic mice, we then asked whether *miR-22* could inhibit these targets in primary cultures of neonatal rat ventricular cardiomyocytes (NRVC). In agreement with a previous report, transfection of a *miR-22* mimic lead to potent hypertrophy of NRVC characterized by increased protein synthesis, cell size, and increased *Nppa* expression (Figures S4A, and ]^14^]). Furthermore, exogenous application of *miR-22* in primary cultures of NRVC confirmed directed repression of PGC-1α, PPARα and SIRT1 (Figure S4B). Collectively, these data establish SIRT1, PGC-1α, and PPARα as bona-fide *miR-22* targets in the heart.

### *miR-22-*mediated cardiomyopathy is associated with impaired ERR/PPAR target gene signature

**Figure 3:**
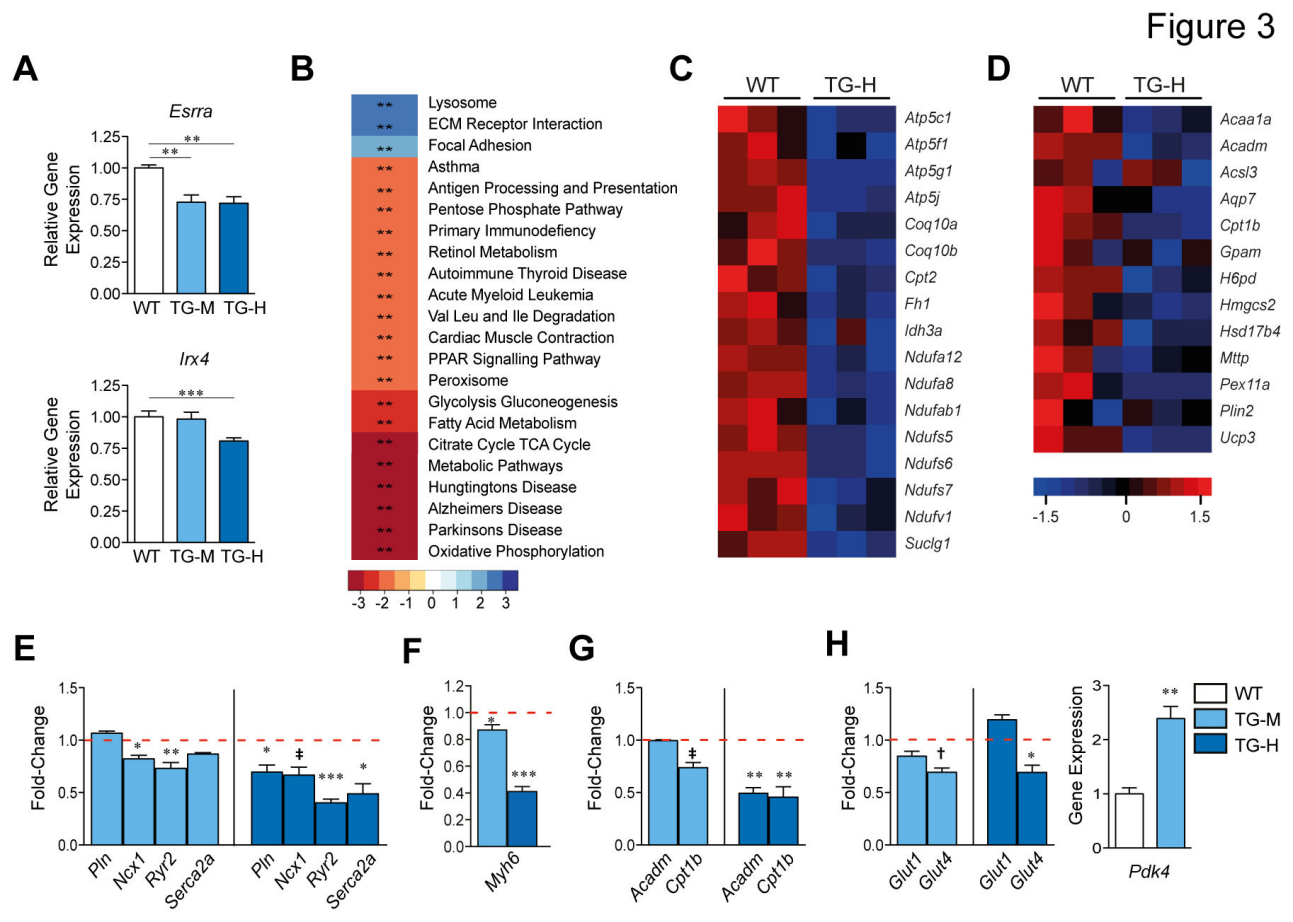
Enforced *miR-22* expression impairs ERR/PPAR-dependent transcription in the heart. (A, E, F, G, and H) Cardiac mRNA expression levels for indicated genes were quantified by qPCR in TG-H, TG-M and WT mice aged (A) 5- or (E-G) 12-weeks old. In panels (E-H) bars represent expression normalized with WT set equal to 1.0 (*n* = 3-4). (B) Gene Set Enrichment Analysis (GSEA) of sorted gene lists obtained from the microarray detected significant expression shifts in the indicated KEGG pathways in TG-H hearts. **, P<0.005. (C and D) Transcriptome microarray-obtained expression profile heat maps for (C) ERR-regulated OXPHOS or (D) PPAR-regulated lipid metabolism genes in 12-week old TG-H and WT mice. Student t test, (E,F,G,H); or 1-way ANOVA with the Dunnett post hoc test, (A). *, P<0.05; **, P<0.01; ***, P<0.001; †, P=0.06; ‡, P=0.07.

We reasoned that hypertrophy and cardiac dysfunction in *miR-22* transgenic mice could be due, at least in part, to decreased expression of SIRT1, PGC1α, and PPARα. Initially, since ERRα is major transcriptional partner of PGC-1α, and PGC-1α is required for ERRα expression/activity itself [7], we wished to determine whether mRNA levels encoding ERRα (*Essra*) were decreased in 5-week old *miR-22* transgenic mice. Secondly, we also scrutinized transcript levels of *Iroquois related homeobox 4* (*Irx4*), a proximal transcriptional brake on hypertrophic traits acting within the PGC-1α/ERR-regulatory axis [10,22]. As shown in Figure 3A, *Esrra* mRNA was repressed in TG-M and TG-H 5-week old hearts and *Irx4* was negatively impacted in the higher line.

Next, we used mRNA microarray combined with real time PCR as a more global approach to determine whether the PGC1α/ERR, PGC1α/PPAR transcription cascades were inhibited by *miR-22* in transgenic mice. As an additional filter, Ingenuity was used to discern unique and combinatorial downstream targets of PGC1α/ERR, and/or PGC1α/PPAR. Microarray GSEA showed coordinate downregulation of genes with KEGG-designated roles in, “cardiac muscle contraction”, “OXPHOS”, “fatty acid metabolism”, “glycolysis”, and “citrate cycle TCA Cycle” in 12-week old TG-H hearts (Figure 3B, Figure S5, and Table S2). A large proportion of impaired OXPHOS and TCA cycle genes [e.g. *NADH dehydrogenase* (*ubiquinone*) *Fe-S* proteins *5* (*Ndufs5*), *NADH dehydrogenase* (*ubiquinone*) *1 alpha subcomplex, 8* (*Ndufa8*), *ATP synthase, H+ transporting, mitochondrial F1 complex, gamma polypeptide 1* (*Atp5c1*)] in 12-week old TG-H hearts are known transcriptional targets of the PGC-1α/ERR complex [10]. We then scrutinized the expression of genes encoding proteins involved in contractile work and/or calcium handling. Using quantitative PCR, we detected lower mRNA amounts of α-cardiac muscle myosin heavy chain (*Myh6*), *Phospholamban* (*Pln*), *Cardiac sodium-calcium exchanger* (*Ncx1*), *Ryanodine receptor 2* (*Ryr2*) and *Sarcoplasmic reticulum Ca* ^*+2*^ *ATPase 2* (*Serca2a*) in TG-H hearts between 5- and 12-weeks of age (Figure 3E, 3F, data not shown and [12]). A similar trend was evident in 12-week old TG-M mice (Figure 3E, 3F). Ingenuity-aided annotation revealed *Myh6*, *Pln, Ryr2*, and *Serca2a* as presumptive targets of PGC-1α/ERR [9,10,22]. We next determined whether cardiac/muscle specific transcription was broadly decreased in *miR-22* transgenic hearts. In agreement with our previous observations, in general muscle-restricted genes encoding proteins in the vicinity of the cardiac Z disc/Titin cytoskeleton appeared either unaffected or induced in *miR-22* transgenic hearts (Figures S3G and [12]).

Next we scrutinized the expression of genes under transcriptional control by PGC-1α/PPARα complex in hearts of *miR-22* transgenic mice. In accordance with expectations, microarray GSEA showed coordinate downregulation of “PPAR signaling pathway” genes in 12-week old TG-H hearts (Figure 3B). These genes included previously validated PPAR-dependent genes [e.g. *Acyl-Coenzyme A dehydrogenase, medium chain* (*Acadm*), *Muscle carnitine palmitoyltransferase 1b* (*Cpt 1b*)*, Uncoupling protein 3* (*Ucp3*)] involved in fatty acid uptake, transport, esterification and/or oxidation (Figure 3D). The mRNA levels encoding ACADM and CPT 1B were less pronounced in TG-H hearts beginning at 5-weeks of age (Figure 3G and data not shown). Cpt *1b* transcript levels appeared repressed in TG-M mice beginning at 12-weeks of age (Figure 3G and data not shown). PPAR/ERR responsive genes involved in glucose transport and oxidation [9,10,22] were also aberrantly expressed in *miR-22* transgenic hearts (Figure 3B, 3H and Table S2).

Although both *miR-22* transgenic mice lines show dosage sensitive negative effects on ERR/PPAR-dependent gene expression, secondary complications associated with cardiomyopathy could not be completely discounted. We therefore determined whether *miR-22* overexpression hampers ERR/PPAR-responsive genes in NRVC. As shown in Figure S4C-F, transfection of *miR-22* into NRVC resulted in lower mRNA abundance of a range of genes influenced by PGC-1α/ERR and/or PGC-1α/PPAR in comparison with cells a control miR-mimic. Application of the TargetScan algorithm did not find mouse/human conserved *miR-22* target sites in other PGC1α, PPARα, and ERRα transcription factor family members. Collectively, this data suggest a pathogenic role of *miR-22* in mediating hypertrophy and cardiac dysfunction by suppressing ERR/PPAR-dependent gene expression via silencing of SIRT1, PGC1α, and PPARα.

### Cardiomyocyte-specific overexpression of *miR-22* impairs calcium handling

**Figure 4:**
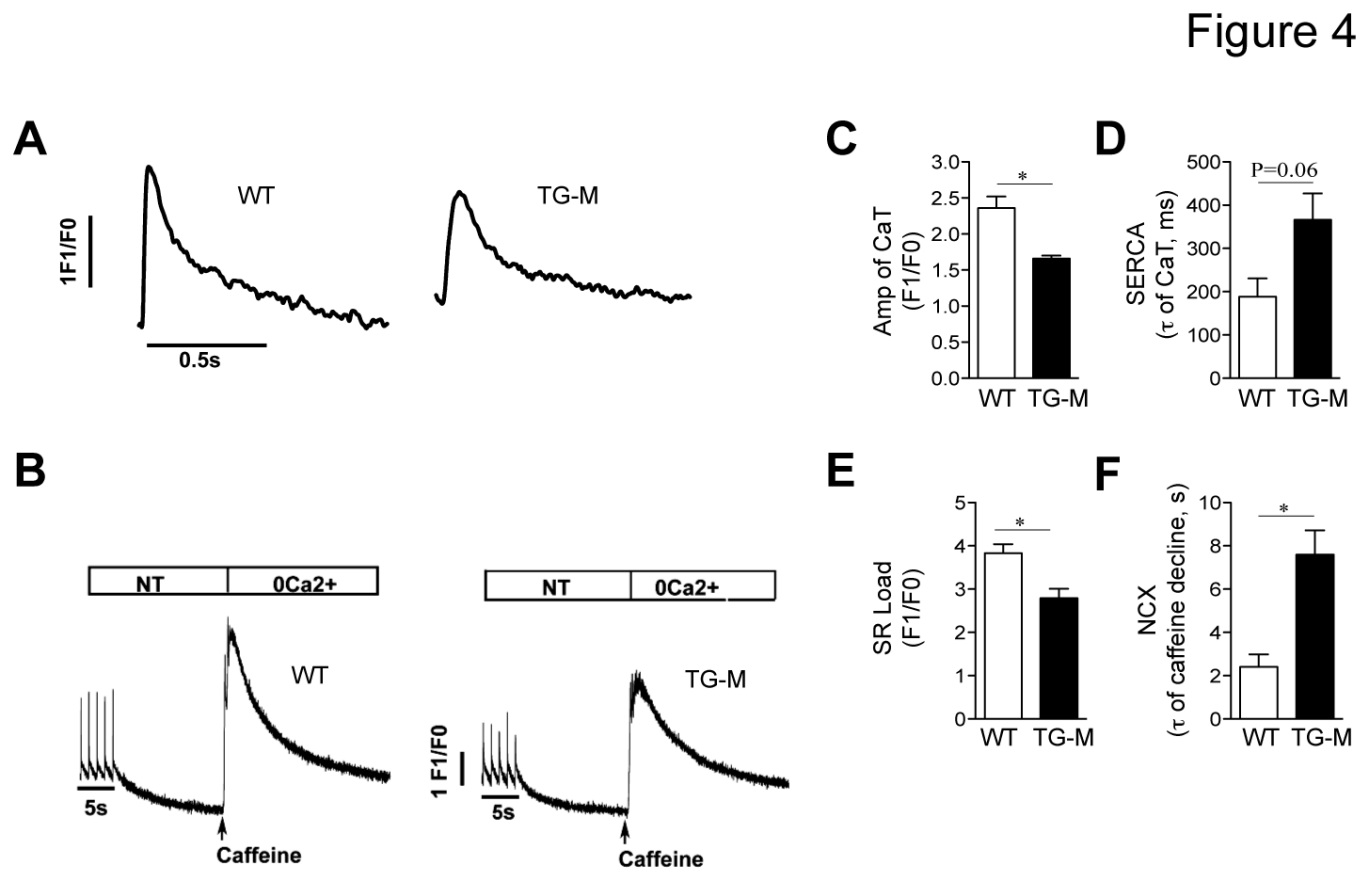
Diminished sarcoplasmic reticulum (SR) Ca^2+^ load and Ca^2+^ transient in *miR-22* transgenic mice. (A) Representative traces of Ca^2+^ transients from TG-M and WT adult ventricular cardiomyocytes upon 1Hz pacing in 1.8mmol/L, Ca^2+^ Tyrode solution, and (B) after rapid exposure to 10mM caffeine. Note that the sweep velocity was constant and the time scale was identical to data in first panel. (C) Quantification of electrically evoked Ca^2+^ transients, and (E) estimation of SERCA2 activity from time constant of Ca^2+^ transient decline (τ of CaT) in TG-M and WT myocytes. (D) SR Ca^2+^ load response, and (F) NCX activity estimation during caffeine stimulation (τ of caffeine decline) in 0 Na^+^, 0 Ca^2+^ Tyrode solution (*n* = 3-4 mice). Student *t* test. *, P<0.05.

The observed alterations in contractile and calcium handling gene expression seen in *miR-22* transgenic mice are predicted to directly affect the magnitude of the Ca^2+^ transient and Ca^2+^ loading in the sarcoplasmic reticulum (SR). To explore this question, we isolated cardiac myocytes from 16-week old TG-M and WT mice and measured calcium transients. When paced at 1Hz, the Ca^2+^ transient amplitude was lower in TG-M compared to WT myocytes (Figure 4A, 4B, 4C). The rate of Ca^2+^ decline following pacing-evoked Ca^2+^ transients revealed a trend towards decreased SERCA2a activity in TG-M mice compared with WT mice (Figure 4D). In addition, measurement of peak Ca^2+^ release following rapid caffeine administration indicated that there was significantly less Ca^2+^ loading in SR in TG-M mice compared to controls (Figure 4E). Quantification of the post-caffeine Ca^2+^ transient decline also revealed reduced Na^+^/Ca^2+^-exchanger (NCX1) activity in TG-M mice compared to WT mice (Figure 4F). Collectively, these results indicate that enhanced *miR-22* expression has a negative effect on calcium handling, which could be the result of impaired SR Ca^2+^ resequestration, suggesting at least in part, a mechanism where overexpression of *miR-22* elicits dysfunction in *miR-22* transgenic mice.

## Discussion

Gain-of-function data presented here clearly shows that *miR-22* is sufficient to induce cardiac hypertrophy and dysfunction and it sheds new light linking overexpression of *miR-22* to ERR/PPAR-dependent transcription. Moderate expression levels of *miR-22* resulted in hypertrophic signaling and an apparent hypocontractile cardiac phenotype. Higher *miR-22* expressing mice exhibited four chamber dilatation, myocardial apoptosis, diastolic ventricular dysfunction and atrial fibrillation. Also, our in-depth molecular analysis revealed a sizable correlation between the *miR-22* transgenic gene signature and published HF related data sets. We also showed that *miR-22* overexpression in cultured neonatal cardiomyocytes promotes classic features of hypertrophy. One interesting aspect of the *miR-22* GOF effect in the heart was the reduced basal contractility seen in TG-M mice in the absence of chamber dilatation and myocardial scarring. Indeed *miR-22* TG-M myocytes also exhibited defective calcium transients, as evidenced by reduced calcium transient amplitudes and diminished SR calcium load. Our interpretation of how enforced *miR-22* expression promotes cardiomyopathy is that *miR-22* proximally compromises contractile function, which then leads to the progressive chamber dilatation and apoptosis as seen in the higher expressing transgenic line.

A plausible mechanism accounting for *miR-22*-mediated GOF cardiomyopathy came from our observation that vital cardiac genes SIRT1, PGC-1α, and PPARα are co-repressed by *miR-22* in the heart. PGC-1α functions upstream of PPARα to maintain the transcription of genes involved in mitochondrial fatty uptake and oxidation [5,7,8]. The activity of PPARα as a transcription factor is dependent on PGC-1α [7,8]. In the heart, PGC-1α is also recruited by ERRα to enhance transcription of ERR target genes [7]. Interestingly, SIRT1 has been reported to positively influence PGC-1α expression and activity at the transcriptional and post-translational level within muscle [23,24]. Moreover, SIRT1 modulates ERR- and PPAR-dependent transcription in a complex manner [22,25]. Therefore, downregulation of SIRT1, PGC-1α, and PPARα expression levels resulting from unchecked *miR-22* cardiac expression may represent a synergistic pathogenic mechanism for elicitation of contractile dysfunction. This interpretation is supported by the molecular signature of enhanced *miR-22* in which ERR and PPAR genes involved in involved in each step of excitation contraction coupling, contractile work, fatty acid/glucose substrate utilization, Krebs cycle activity, ATP synthesis by OXPHOS and hypertrophy gene transcription are downregulated in the heart. This molecular signature of enhanced *miR-22* is reminiscent of PGC-1α or ERRα gene disruption [9,10,19]. The central regulators of Ca^2+^ transients and contractile work, PLN, RYR2, SERCA2a and α-MHC respectively are positively influenced, at least in part, at the level of transcription by the PGC1α/ERR regulatory cascade [9,10,22]. Consequently, at one level, enforced *miR-22* expression levels may cause a decline in the transcription of these genes culminating in impaired calcium handling and pump dysfunction. These results were confirmed, indeed *miR-22* transgenic myocytes exhibited defective calcium transients, as evidenced by reduced calcium transient amplitudes and diminished SR calcium load. A limitation of our studies is that they did not address whether deranged ERR and PPAR transcription in *miR-22* transgenic hearts are associated with ATP-depletion, mitochondrial dysfunction, and/or abnormal substrate utilization.

We have previously shown that absence of *miR-22* also renders the heart sensitive to dilatation and decompensation with stress provocation [12]. Our molecular analysis in the *miR-22* LOF model suggested that *miR-22* works, in large part, within myocytes to sustain maximal expression levels of virtually all muscle-restricted serum response factor-dependent genes. Its worthy of note that *miR-22* has been reported to be both up- and down-regulated in human heart disease [26–28]. This would suggest that *miR-22* functions within a tight threshold in the heart. It should be recognized that the mechanism(s) of *miR-22* LOF and GOF mediated cardiomyopathy are especially complex because *miR-22* operates upstream of many critical transcription factors with a high level of connectivity and therefore the phenotype(s) cannot be easily be assigned to a single gene. In the future it will be important to determine if *miR-22* is inversely correlated with expression levels of SIRT1, PGC-1α, and PPARα in human diseased hearts.

## Supporting Information

Figure S1 **Representative Doppler Lead II surface electrocardiogram trace from 5-week old TG-H and WT mice.**
(TIF)

Figure S2 **Apoptosis analysis in *miR-22* transgenic mice.** Histomorphometric quantification of TUNEL-positive cells in cardiac sections obtained from 12-week old TG-H and control mice. (*n* = 3-4.) Student *t* test. **, P<0.01.
(TIF)

Figure S3 **Transcriptome microarray detection of *miR-22* targeting effects.** (A) Sylamer analysis of microarray shows specific enrichment of *miR-22* heptamer ‘seed’ matches among downregulated genes in TG-H hearts. *miR-22* seed heptamers are shown in shades of blue while all other mouse miRNA seeds are shown in grey. (B) Pie chart shows the number and distribution of downregulated transcripts in TG-H hearts with 6-9-mer seed matches in 3’ UTR. (C and E) Relative cardiac expression levels of *miR-22* target genes were evaluated by qPCR in (E) 5- or (C) 12-week old WT, TG-M, and TG-H mice. (D) CAV3 protein levels were determined by immunoblot in hearts of 12-week old TG-H and WT mice. (F) Schematic shows conserved motifs potentially bound by *miR-22* in the 3’ UTRs of *Camta2* and *Homer1*. (G) Quantitative PCR detection of sarcomeric genes in 12-week old TG-M versus WT hearts. Bars represent expression normalized with WT set equal to 1.0. Student *t* test, (C and G); or 1-way ANOVA with the Dunnett post hoc test, (E). *, P<0.05; **, P<0.01; ***, P<0.001.
(TIF)

Figure S4 **Cellular and molecular effects of increased *miR-22* expression in primary cultures of neonatal rat ventricular cardiac myocytes (NRVC).** (A) Representative α-actinin immunostaining and cardiomyocyte cell size of NRVC transfected with either a *miR-22* or control (Crtl) miRNA mimic in the absence (Basal) or presence of phenylephrine (PE); *n* = 3-5 experiments, >40,000 cells per group. (B) Immunoblot detection of PGC-1α, PPARα, and SIRT1 levels in NRVC cells transfected with either a *miR-22* or control miRNA mimic. GAPDH was used as a loading control. Repression: protein levels in *miR-22* over-expressing cells relative to control miRNA. (C, D, E, F) Real time quantitative PCR detection of indicated genes in NRVC cells transfected with *miR-22* or control miR mimic (n = 3 experiments). Student *t* test, (C, D, E, F); or 2-way ANOVA with the Tukey’ post hoc test, (A) *, P<0.05; **, P<0.01.
(TIF)

Figure S5 **Transcriptome microarray Gene Set Enrichment Analysis.** Gene set enrichment analysis (GSEA) tool was applied on the transcriptome microarray to identify significantly repressed or induced Gene Ontology, Biological Process, Molecular Function, or Cellular Compartment categories in *miR-22* transgenic hearts. *, P<0.05; **, P<0.005.
(TIF)

Table S1 **Hemodynamic Analysis of TG-H mice and WT controls.**
(PDF)

Table S2 **Genes Dysregulated in miR-22 transgenic hearts.**
(PDF)

Table S3 **Potential Targets of miR-22 in the heart.**
(PDF)
