# Supplementary figures and images for: microRNA-22 Promotes Heart Failure through Coordinate Suppression of PPAR/ERR-Nuclear Hormone Receptor Transcription
Source: PLoS One. 2013 Sep 27;8(9):e75882. doi: 10.1371/journal.pone.0075882 (PMC3785418; doi:10.1371/journal.pone.0075882)

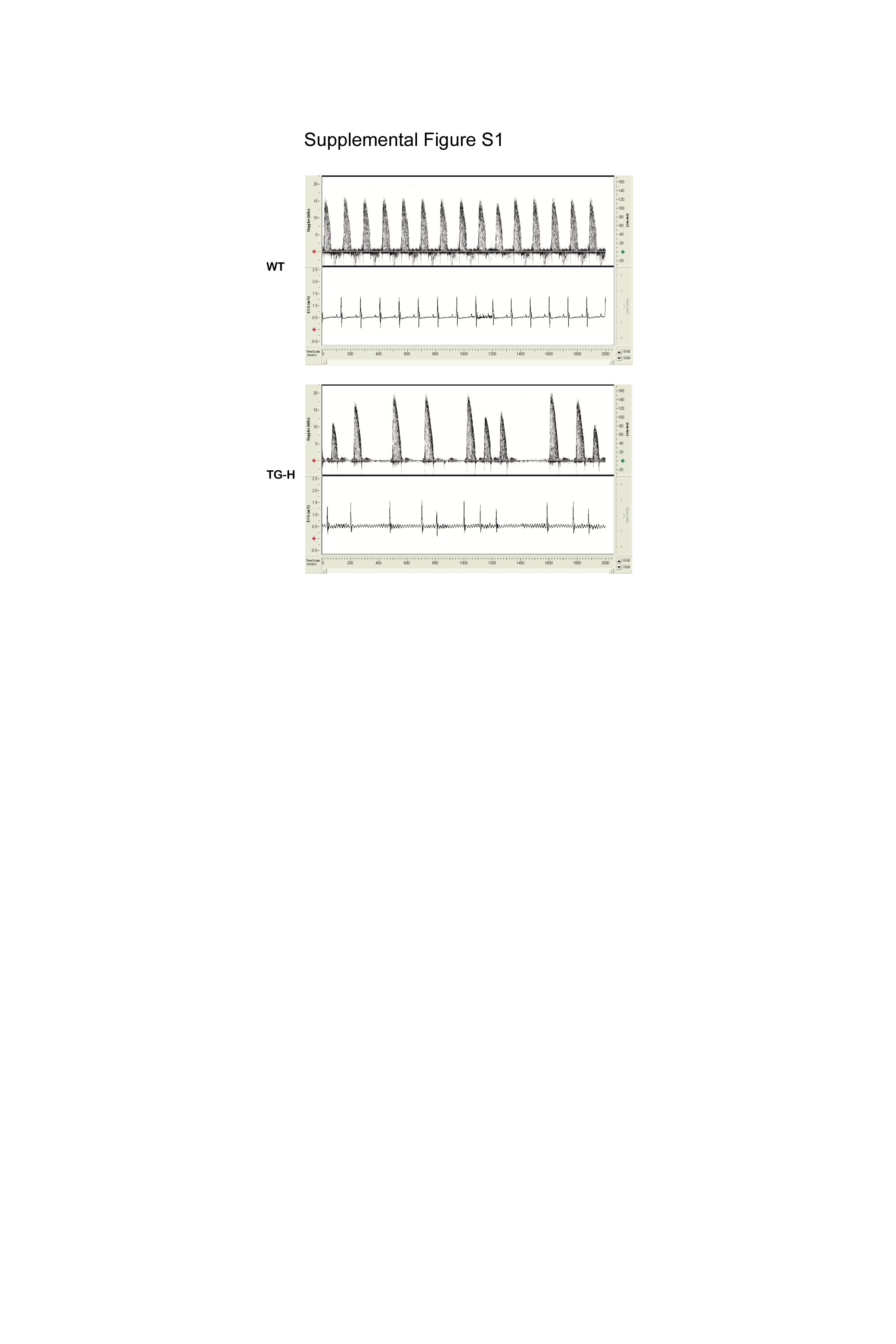

Supplement: Figure S1 — Representative Doppler Lead II surface electrocardiogram trace from 5-week old TG-H and WT mice. (TIF) [file pone.0075882.s001.tif]

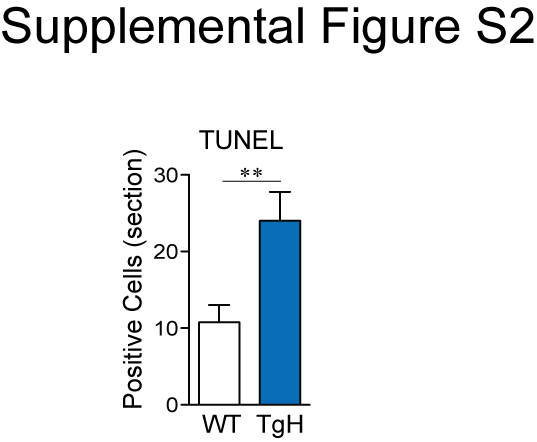

Supplement: Figure S2 — Apoptosis analysis in miR-22 transgenic mice. Histomorphometric quantification of TUNEL-positive cells in cardiac sections obtained from 12-week old TG-H and control mice. (n = 3-4.) Student t test. **, P<0.01. (TIF) [file pone.0075882.s002.tif]

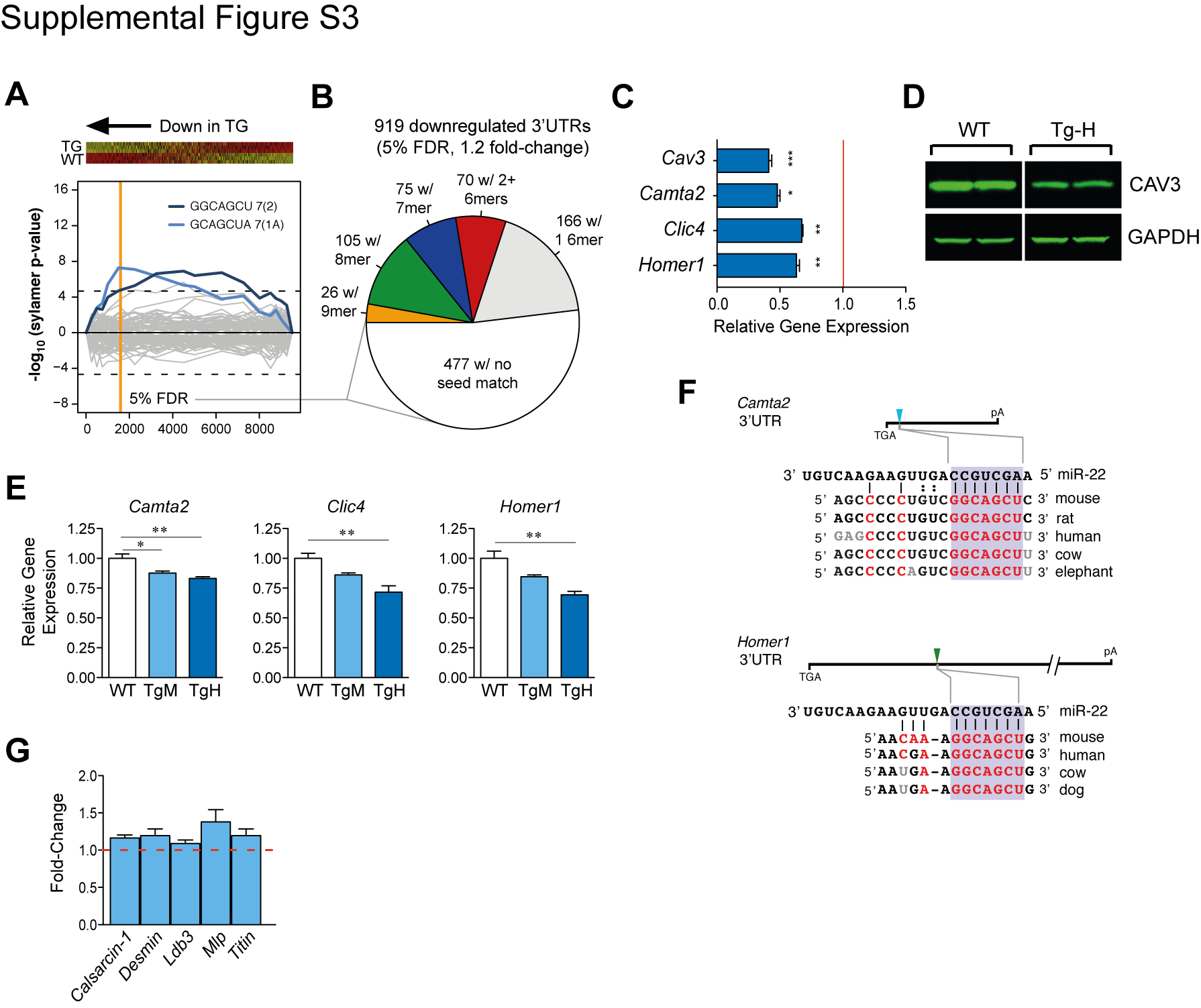

Supplement: Figure S3 — Transcriptome microarray detection of miR-22 targeting effects. (A) Sylamer analysis of microarray shows specific enrichment of miR-22 heptamer ‘seed’ matches among downregulated genes in TG-H hearts. miR-22 seed heptamers are shown in shades of blue while all other mouse miRNA seeds are shown in grey. (B) Pie chart shows the number and distribution of downregulated transcripts in TG-H hearts with 6-9-mer seed matches in 3’ UTR. (C and E) Relative cardiac expression levels of miR-22 target genes were evaluated by qPCR in (E) 5- or (C) 12-week old WT, TG-M, and TG-H mice. (D) CAV3 protein levels were determined by immunoblot in hearts of 12-week old TG-H and WT mice. (F) Schematic shows conserved motifs potentially bound by miR-22 in the 3’ UTRs of Camta2 and Homer1. (G) Quantitative PCR detection of sarcomeric genes in 12-week old TG-M versus WT hearts. Bars represent expression normalized with WT set equal to 1.0. Student t test, (C and G); or 1-way ANOVA with the Dunnett post hoc test, (E). *, P<0.05; **, P<0.01; ***, P<0.001. (TIF) [file pone.0075882.s003.tif]

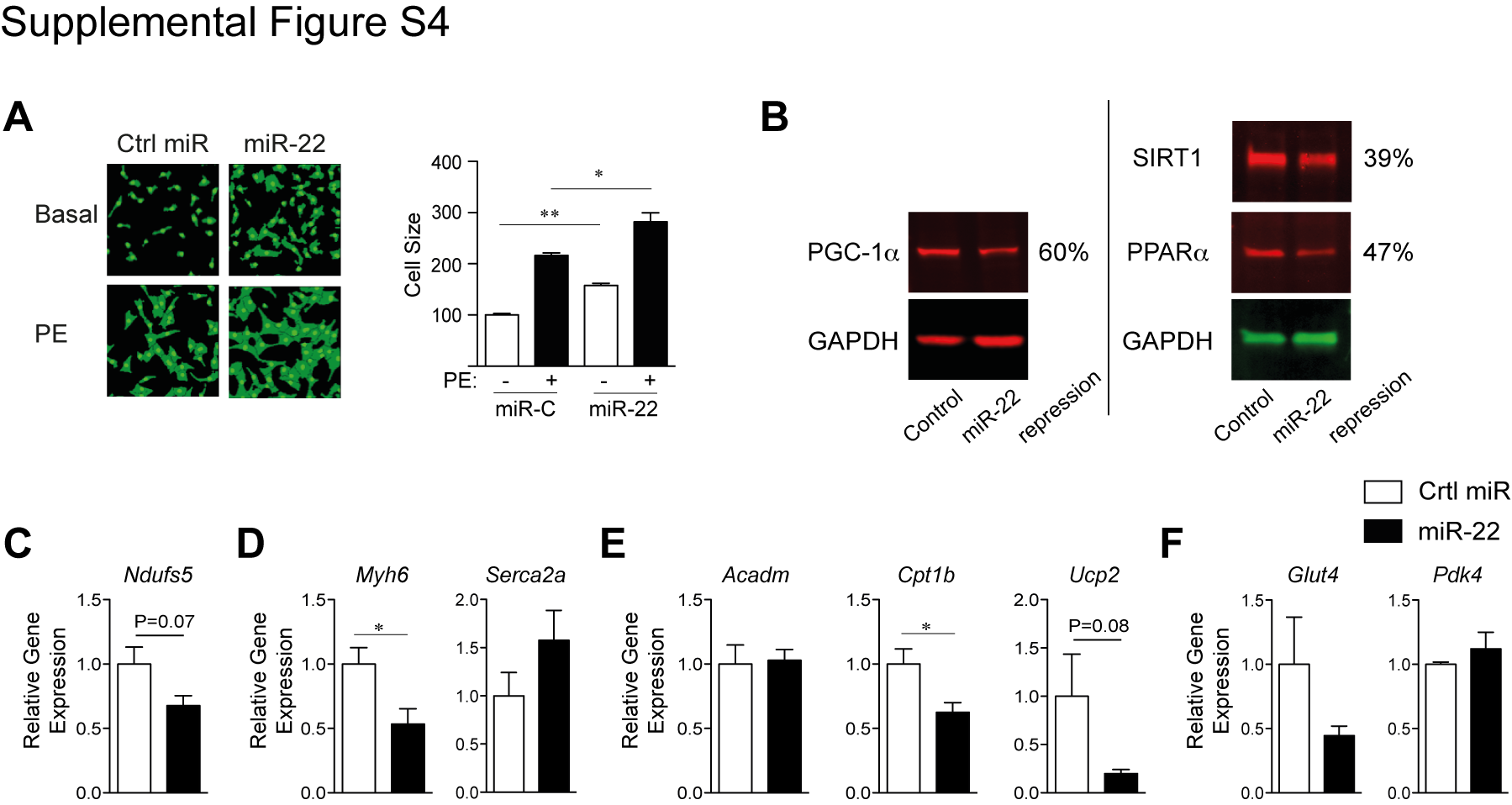

Supplement: Figure S4 — Cellular and molecular effects of increased miR-22 expression in primary cultures of neonatal rat ventricular cardiac myocytes (NRVC). (A) Representative α-actinin immunostaining and cardiomyocyte cell size of NRVC transfected with either a miR-22 or control (Crtl) miRNA mimic in the absence (Basal) or presence of phenylephrine (PE); n = 3-5 experiments, >40,000 cells per group. (B) Immunoblot detection of PGC-1α, PPARα, and SIRT1 levels in NRVC cells transfected with either a miR-22 or control miRNA mimic. GAPDH was used as a loading control. Repression: protein levels in miR-22 over-expressing cells relative to control miRNA. (C, D, E, F) Real time quantitative PCR detection of indicated genes in NRVC cells transfected with miR-22 or control miR mimic (n = 3 experiments). Student t test, (C, D, E, F); or 2-way ANOVA with the Tukey’ post hoc test, (A) *, P<0.05; **, P<0.01. (TIF) [file pone.0075882.s004.tif]

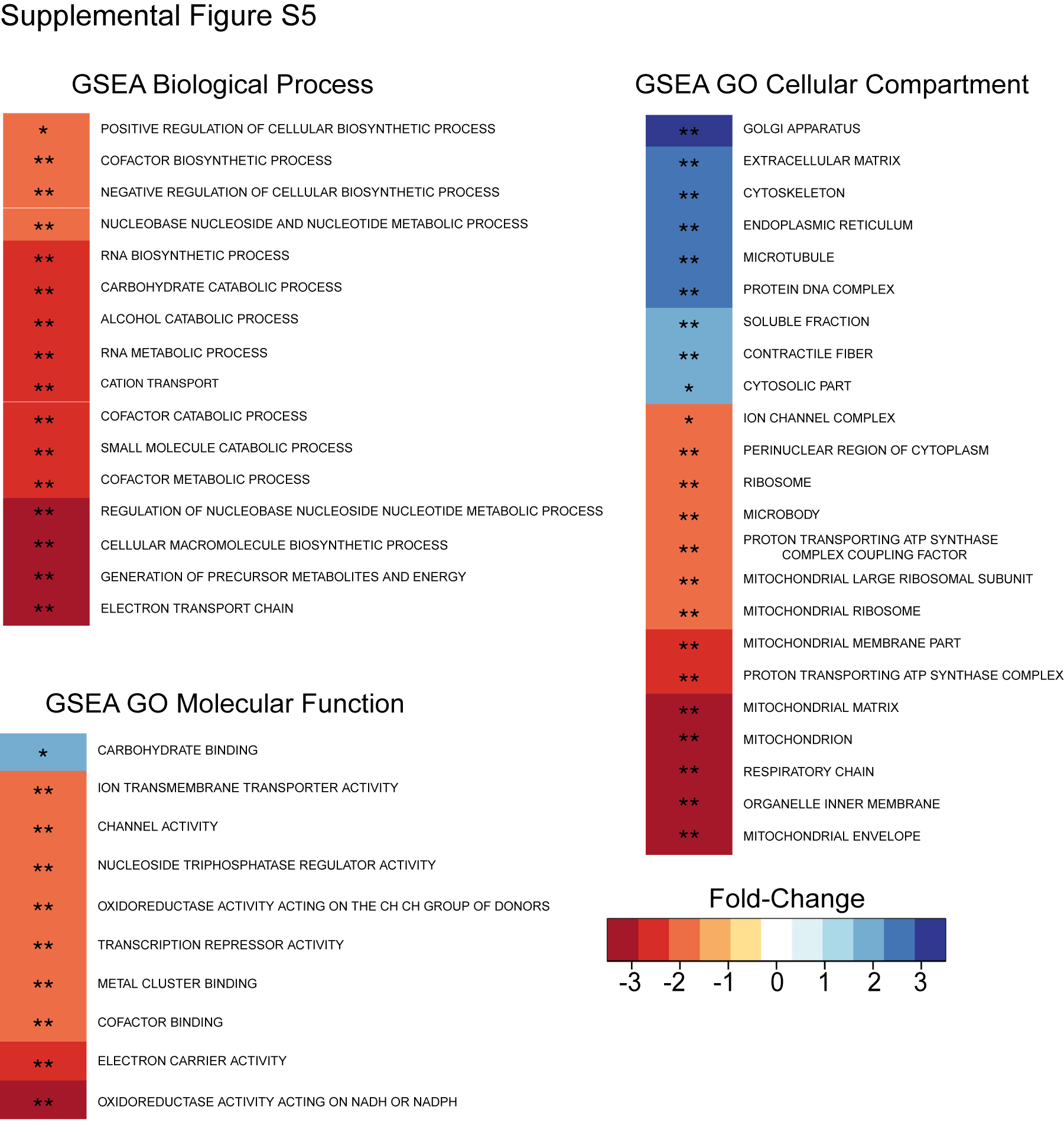

Supplement: Figure S5 — Transcriptome microarray Gene Set Enrichment Analysis. Gene set enrichment analysis (GSEA) tool was applied on the transcriptome microarray to identify significantly repressed or induced Gene Ontology, Biological Process, Molecular Function, or Cellular Compartment categories in miR-22 transgenic hearts. *, P<0.05; **, P<0.005. (TIF) [file pone.0075882.s005.tif]
