# Supplementary material for: microRNA-22 Promotes Heart Failure through Coordinate Suppression of PPAR/ERR-Nuclear Hormone Receptor Transcription
Source: PLoS One. 2013 Sep 27;8(9):e75882. doi: 10.1371/journal.pone.0075882 (PMC3785418; doi:10.1371/journal.pone.0075882)
Supplement: Table S1 — Hemodynamic Analysis of TG-H mice and WT controls. (PDF) [file pone.0075882.s006.pdf]

Table S1. Hemodynamic analysis of TG-H mice and WT controls

	8-weeks old	
	**WT**	**TG-H**
n	4-7	5-6
HR (bpm)	424 ± 32	**324 ± 68***
LA vol. (mm^3)	4.60 ± 0.50	**11.10 ± 0.70*****
LVPWS (mm)	1.11 ± 0.08	0.99 ± 0.07
LVEDD (mm)	3.26 ± 0.13	**4.00 ± 0.20***
FS (%)	49.0 ± 3.60	41.0 ± 1.50
IVRT (msec)	13.71 ± 0.5	**19.9 ± 1.2****
PAoFV (cm/sec)	112.3 ± 7	109.0 ± 8
PEV (cm/sec)	83 ± 6	96 ± 4
CVR-R (%)	3.8 ± 1.1	**44.8 ± 16.5***

HR, heart rate; LA vol, Left Atrial volume; LVPWS, Left Ventricular (LV) end-systolic posterior wall thickness; LVEDD, LV end-diastolic dimension; FS, fractional shortening; IVRT, Isovolumic relaxation time; PAoFV, Peak Aortic Flow Velocity; PEV, Peak Ejection Flow Velocity; CVR-R, Coefficient of Variation of R-R Intervals. Data were analyzed by Student *t*-test. *, $P < 0.05$; **, $P < 0.01$; ***, $P < 0.005$ versus WT.
