# Supplementary material for: microRNA-22 Promotes Heart Failure through Coordinate Suppression of PPAR/ERR-Nuclear Hormone Receptor Transcription
Source: PLoS One. 2013 Sep 27;8(9):e75882. doi: 10.1371/journal.pone.0075882 (PMC3785418; doi:10.1371/journal.pone.0075882)
Supplement: Table S2 — Genes Dysregulated in miR-22 transgenic hearts. (PDF) [file pone.0075882.s007.pdf]

Table S2. Genes disregulated in *miR-22*-transgenic hearts

Down-regulated genes (genes with greater or equal to 1.5-fold repression are shown)

Symbol	Description	FC	P.Value	adj.P.Val	Transcript	3'UTR_length	9mers	8mers	7mers	6mers	9mers_cons	8mers_cons	7mers_cons	6mers_cons	TS6_PCT	TSC_Context Plus_rerun	TargetScan_ORF
Ano10	anoctamin 10	-5.11	2.12E-08	4.82E-06	ENSMUST00000042546	534	0	0	0	0							
scl000994.1_0		-4.97	3.52E-08	6.98E-06	AK052936	224	0	0	0	0							
Ces3	carboxylesterase 3	-4.49	5.71E-09	2.96E-06	OTTMUST00000060241	187	0	0	0	0							
Fhl2	four and a half LIM domains 2	-4.38	6.10E-07	3.39E-05	ENSMUST00000008280	440	0	0	0	1				0.682			
Whrn	whirlin	-3.29	2.72E-08	5.89E-06	OTTMUST00000000708	574	0	0	0	0							
Lrtm1	leucine-rich repeats and transmembrane domains 1	-2.85	1.47E-06	5.88E-05	ENSMUST00000055662	126	0	0	0	0							
Scn4b	sodium channel, type IV, beta	-2.82	1.14E-06	5.01E-05	OTTMUST00000040454	3648	0	0	1	2			-0.13	-0.117		-0.107	
Bdh1	3-hydroxybutyrate dehydrogenase, type 1	-2.79	6.91E-09	3.31E-06	OTTMUST00000063795	1807	0	0	0	1				0.904			
Gpr22	G protein-coupled receptor 22	-2.58	5.30E-08	8.55E-06	OTTMUST00000054817	1844	0	0	0	2				0.023			
P2ry1	purinergic receptor P2Y, G-protein coupled 1	-2.54	7.46E-10	1.16E-06	ENSMUST00000029331	2117	0	0	0	1				1.551			
Eno3	enolase 3, beta muscle	-2.51	2.27E-06	7.93E-05	OTTMUST00000013691	81	0	0	0	0							
Pdp2	pyruvate dehyrogenase phosphatase catalytic subunit 2	-2.48	2.11E-07	1.84E-05	ENSMUST00000059588	1717	0	0	1	0			-0.08			-0.095	
Acot1	acyl-CoA thioesterase 1	-2.48	2.60E-05	0.000412	ENSMUST00000085210	292	0	0	0	0							
Apbb1	amyloid beta (A4) precursor protein-binding, family B, member 1	-2.41	2.76E-08	5.89E-06	ENSMUST00000081165	397	0	0	0	0							
Ky	kyphoscoliosis peptidase	-2.4	3.25E-08	6.57E-06	ENSMUST00000039390	3457	0	0	0	0							
Igtp	interferon gamma induced GTPase	-2.4	0.011	0.0348	OTTMUST00000012730	315	0	0	0	0							
Cish	cytokine inducible SH2-containing protein	-2.33	8.02E-06	0.00019	ENSMUST00000085102	1061	0	0	0	0							
Pdlim4	PDZ and LIM domain 4	-2.25	5.44E-07	3.20E-05	OTTMUST00000012588	221	0	0	0	0							
Ppil1	peptidylprolyl isomerase (cyclophilin)-like 1	-2.24	5.60E-10	1.12E-06	OTTMUST00000071754	702	0	0	0	0							
Kcnv2	potassium channel, subfamily V, member 2	-2.21	7.20E-08	9.78E-06	OTTMUST00000048473	3256	0	1	0	2		0.155		0.12		-0.164	
Snrpn	small nuclear ribonucleoprotein N	-2.2	3.21E-09	2.74E-06	ENSMUST00000059305	738	0	0	0	0							
Myh6	myosin, heavy polypeptide 6, cardiac muscle, alpha	-2.2	6.08E-06	0.000158	ENSMUST00000081857	52	0	0	0	0							
Mlxipl	MLX interacting protein-like	-2.19	2.94E-08	6.06E-06	OTTMUST00000064065	1022	0	0	0	1				-0.559			
Fam78a	family with sequence similarity 78, member A	-2.17	5.92E-07	3.34E-05	OTTMUST00000031096	2378	0	0	0	1				-0.733			
Ttll1	tubulin tyrosine ligase-like 1	-2.16	9.59E-08	1.16E-05	OTTMUST00000057014	412	0	0	0	0							
Egfbp2	epidermal growth factor binding protein type B	-2.14	1.92E-08	4.53E-06	ENSMUST00000048945	41	0	0	0	0							
Ggnbp1	gametogenetin binding protein 1	-2.13	6.83E-08	9.55E-06	OTTMUST00000076818	212	0	0	0	0							
Dhrs11	dehydrogenase/reductase (SDR family) member 11	-2.11	3.87E-08	7.12E-06	OTTMUST00000001927	404	0	0	0	1				0.312			
Fkbp4	FK506 binding protein 4	-2.11	1.80E-06	6.72E-05	OTTMUST00000060562	673	0	0	0	1				0.574			
Gpc1	glypican 1	-2.11	9.25E-06	0.000211	ENSMUST00000045970	1634	0	2	0	2		-0.346		-0.863		-0.212	
Dci	dodecenoyl-Coenzyme A delta isomerase (3,2 trans-enoyl-Coenyme A isomerase)	-2.1	1.77E-06	6.64E-05	ENSMUST00000024946	201	0	0	0	0							
Cd74	CD74 antigen (invariant polypeptide of major histocompatibility complex, class II antigen-associated)	-2.1	0.0042	0.0165	ENSMUST00000050487	484	0	0	0	0							
Ccbl1	cysteine conjugate-beta lyase 1	-2.09	1.36E-08	3.80E-06	OTTMUST00000040651	380	0	0	0	0							
Maob	monoamine oxidase B	-2.08	9.63E-06	0.000217	OTTMUST00000041060	757	0	0	0	0							
Rpl3l	ribosomal protein L3-like	-2.07	3.96E-05	0.000537	ENSMUST00000045186	89	0	0	0	0							
Dgat2	diacylglycerol O-acyltransferase 2	-2.06	6.31E-08	9.55E-06	ENSMUST00000033001	888	0	0	0	0							
Cyp2b10	cytochrome P450, family 2, subfamily b, polypeptide 10	-2.06	3.10E-05	0.000463	OTTMUST00000067194	375	0	0	0	0							
Foxred1	FAD-dependent oxidoreductase domain containing 1	-2.04	4.69E-08	7.87E-06	OTTMUST00000086277	661	0	1	0	0		-0.502				-0.289	
Fndc5	fibronectin type III domain containing 5	-2.04	6.19E-07	3.42E-05	OTTMUST00000022138	2053	0	0	1	3			0.031	2.112		-0.076	
Gzmm	granzyme M (lymphocyte met-ase 1)	-2.04	9.09E-07	4.28E-05	OTTMUST00000069005	101	0	0	0	0							
Ptp4a3	protein tyrosine phosphatase 4a3	-2.01	1.13E-08	3.41E-06	ENSMUST00000053232	312	0	0	0	0							
Tmem25	transmembrane protein 25	-2	2.02E-07	1.81E-05	OTTMUST00000040541	1199	0	0	0	1				0.348			
LOC641240	histocompatibility antigen, A-D	-2	0.0132	0.0401	XM_918601	311	0	0	0	0							
Tbc1d10c	TBC1 domain family, member 10c	-1.99	3.70E-07	2.49E-05	ENSMUST00000045864	368	0	0	0	0							
A2bp1	ataxin 2 binding protein 1	-1.96	2.11E-05	0.000356	ENSMUST00000115841	1697	0	0	0	0							
Aldh5a1	aldhehyde dehydrogenase family 5, subfamily A1	-1.95	5.33E-08	8.55E-06	OTTMUST00000001200	3982	0	0	1	3			1.359	-1.528		-0.15	
Tmem82	transmembrane protein 82	-1.95	3.08E-07	2.22E-05	OTTMUST00000024087	348	0	0	0	0							
Dbh	dopamine beta hydroxylase	-1.93	6.68E-06	0.000169	OTTMUST00000027810	400	0	0	0	0							
St3gal5	ST3 beta-galactoside alpha-2,3-sialyltransferase 5	-1.88	7.14E-07	3.67E-05	ENSMUST00000069994	900	0	1	2	2		-0.798	1.988	0.101		-0.502	
H2-T22	histocompatibility 2, T region locus 22	-1.88	0.0043	0.0168	OTTMUST00000035506	344	0	0	0	0							
H2-Ab1	histocompatibility 2, class II antigen A, beta 1	-1.87	0.0107	0.0341	ENSMUST00000040828	302	0	0	0	0							
Mtr	5-methyltetrahydrofolate-homocysteine methyltransferase	-1.86	3.15E-06	0.000101	ENSMUST00000099856	469	0	0	0	0							
Ckm	creatine kinase, muscle	-1.85	1.49E-08	3.95E-06	ENSMUST00000003643	197	0	0	0	0							
Acaa2	acetyl-Coenzyme A acyltransferase 2 (mitochondrial 3-oxoacyl-Coenzyme A thiolase)	-1.85	1.38E-05	0.000268	ENSMUST00000041053	268	0	0	0	0							
Ybx2	Y box protein 2	-1.84	4.11E-07	2.70E-05	OTTMUST00000013542	414	0	0	0	0							
Tmem50b	transmembrane protein 50B	-1.84	4.25E-07	2.76E-05	OTTMUST00000047595	1610	0	1	0	1		1.598		1.216		-0.137	
Fbp2	fructose bisphosphatase 2	-1.84	0.000713	0.00432	ENSMUST00000021907	202	0	0	0	0							
Dnaja3	DnaJ (Hsp40) homolog, subfamily A, member 3	-1.83	1.43E-08	3.90E-06	OTTMUST00000059741	1141	0	1	0	2		-0.169		0.983		-0.201	
Pcp4l1	Purkinje cell protein 4-like 1	-1.83	7.06E-06	0.000176	OTTMUST00000051423	1121	0	0	0	1				0.505			
Bcl11b	B-cell leukemia/lymphoma 11B	-1.82	4.36E-07	2.77E-05	OTTMUST00000046516	4997	0	0	0	1				0.15			
Mmp15	matrix metallopeptidase 15	-1.82	3.43E-05	0.000491	OTTMUST00000067975	1773	0	0	0	2				1			
Mrps9	mitochondrial ribosomal protein S9	-1.81	3.56E-07	2.43E-05	ENSMUST00000057208	184	0	0	0	0							
1810006K21Rik	RIKEN cDNA 1810006K21 gene	-1.8	8.94E-09	3.31E-06	OTTMUST00000086010	118	0	1	0	0		0.256				-0.232	
Timm44	translocase of inner mitochondrial membrane 44	-1.79	8.62E-08	1.11E-05	OTTMUST00000052863	405	0	1	1	0		-1.767	-1.15			-0.529	

Symbol	Description	FC	P.Value	adj.P.Val	Transcript	3'UTR_length	9mers	8mers	7mers	6mers	9mers_cons	8mers_cons	7mers_cons	6mers_cons	TS6_PCT	TSC_Context Plus_rerun	TargetScan_ORF
Gpt	glutamic pyruvic transaminase, soluble	-1.79	1.10E-05	0.000231	ENSMUST00000023203	164	0	0	0	0							
H2-D1	histocompatibility 2, D region locus 1	-1.79	0.00268	0.0117	ENSMUST00000087173	625	0	0	0	2							
Hcn2	hyperpolarization-activated, cyclic nucleotide-gated K+ 2	-1.78	2.07E-07	1.84E-05	OTTMUST00000068991	568	0	0	0	0							
Mrps12	mitochondrial ribosomal protein S12	-1.78	4.32E-07	2.76E-05	ENSMUST00000056078	227	0	0	0	0							
Rnf207	ring finger protein 207	-1.78	0.00014	0.00131	OTTMUST00000024276	408	0	0	0	0							
Rmnd1	required for meiotic nuclear division 1 homolog (S. cerevisiae)	-1.77	3.36E-09	2.74E-06	OTTMUST00000076631	1554	0	0	0	1							
Pdss1	prenyl (solanesyl) diphosphate synthase, subunit 1	-1.77	9.71E-08	1.16E-05	OTTMUST00000040189	303	1	0	0	0	2.166					-0.597	
Nudt4	nudix (nucleoside diphosphate linked moiety X)-type motif 4	-1.76	4.08E-09	2.78E-06	ENSMUST00000020217	1999	0	1	0	1		1.081		0.475		-0.448	
Dpy30	dpy-30 homolog (C. elegans)	-1.76	5.57E-07	3.23E-05	ENSMUST00000112571	271	0	1	0	0						-0.31	
Naprt1	nicotinate phosphoribosyltransferase domain containing 1	-1.76	2.71E-06	9.05E-05	ENSMUST00000023237	31	0	0	0	0							
Acsl6	acyl-CoA synthetase long-chain family member 6	-1.76	2.18E-05	0.000365	OTTMUST00000012611	3559	0	0	0	2				0.865			
Tmem223	transmembrane protein 223	-1.76	0.000157	0.00143	ENSMUST00000010248	135	0	0	0	0							
Acadvl	acyl-Coenzyme A dehydrogenase, very long chain	-1.75	2.77E-06	9.17E-05	OTTMUST00000013531	113	0	0	0	0							
Plin5	perilipin 5	-1.75	2.02E-05	0.000347	ENSMUST00000019808	428	0	0	0	0							
Retnla	resistin like alpha	-1.74	5.32E-05	0.000656	ENSMUST00000023329	160	0	0	0	0							
Slc46a3	solute carrier family 46, member 3	-1.73	6.80E-08	9.55E-06	OTTMUST00000071250	696	0	0	0	0							
BC022713	cDNA sequence BC022713	-1.73	1.30E-07	1.43E-05	NM_177761												
Nat5	N-acetyltransferase 5 (ARD1 homolog, S. cerevisiae)	-1.73	1.95E-07	1.80E-05	OTTMUST00000009059	536	1	0	0	0	0.592					-0.54	
Car14	carbonic anhydrase 14	-1.73	1.98E-07	1.81E-05	OTTMUST00000052679	378	0	0	0	0							
Macrod1	MACRO domain containing 1	-1.73	4.28E-06	0.000122	ENSMUST00000040261	175	0	0	0	0							
Josd2	Josephin domain containing 2	-1.72	3.52E-09	2.74E-06	OTTMUST00000071936	138	0	0	0	1				0.11			
Smtn	smoothelin	-1.72	4.35E-05	0.00058	OTTMUST00000011165	325	0	0	0	0							
Ppbp	pro-platelet basic protein	-1.72	0.00466	0.0178	ENSMUST00000031319	653	0	0	0	0							
Tesc	tescalcin	-1.71	1.61E-07	1.66E-05	OTTMUST00000050483	199	0	0	0	0							
Dhrs7c	dehydrogenase/reductase (SDR family) member 7C	-1.71	1.38E-06	5.70E-05	OTTMUST00000013210	52	0	0	0	0							
Gck	glucokinase	-1.71	2.54E-06	8.64E-05	OTTMUST00000014370	850	0	1	0	1		-0.554		-1.769		-0.243	
Adhfe1	alcohol dehydrogenase, iron containing, 1	-1.7	9.46E-08	1.16E-05	OTTMUST00000053148	1696	1	0	0	0						-0.404	
Rasd2	RASD family, member 2	-1.7	1.44E-07	1.54E-05	OTTMUST00000085986	1865	0	0	0	2							
Gm14492	predicted gene 14492	-1.7	8.45E-07	4.16E-05	OTTMUST00000040867	732	0	0	0	0							
Fitm1	fat storage-inducing transmembrane protein 1	-1.7	3.10E-05	0.000463	ENSMUST00000022826	25	0	0	0	0							
Gm10499	predicted gene 10499	-1.7	0.00212	0.0098	ENSMUST00000064686	211	0	0	0	1							
2410015M20Rik	RIKEN cDNA 2410015M20 gene	-1.69	1.70E-06	6.44E-05	ENSMUST00000052832	122	0	0	1	0						-0.199	
2810416G20Rik	RIKEN cDNA 2810416G20 gene	-1.69	3.98E-06	0.000118	ENSMUST00000057404												
Cav3	caveolin 3	-1.69	2.04E-05	0.000348	ENSMUST00000075477	392	1	0	0	0	1.92					-0.463	
Inmt	indolethylamine N-methyltransferase	-1.69	0.00207	0.00961	OTTMUST00000066722	222	0	0	0	0							
Ech1	enoyl coenzyme A hydratase 1, peroxisomal	-1.68	0.000468	0.00315	OTTMUST00000077226	139	0	0	0	0							
Tnfaip8	tumor necrosis factor, alpha-induced protein 8	-1.67	9.59E-09	3.31E-06	OTTMUST00000083080	1017	0	0	0	0							
Dhx30	DEAH (Asp-Glu-Ala-His) box polypeptide 30	-1.66	1.77E-08	4.38E-06	OTTMUST00000086417	73	0	0	0	0							
2610528E23Rik	RIKEN cDNA 2610528E23 gene	-1.66	1.68E-06	6.42E-05	ENSMUST00000023430	199	0	0	0	0							
Tomm6	translocase of outer mitochondrial membrane 6 homolog (yeast)	-1.66	5.92E-06	0.000155	OTTMUST00000044344	367	0	1	0	0		2.01				-0.249	
Snai3	snail homolog 3 (Drosophila)	-1.66	0.000258	0.00205	ENSMUST00000006762	645	0	0	1	1			-0.47			-0.164	
Cpped1	Vcalcineurin-like phosphoesterase domain containing 1	-1.65	6.53E-08	9.55E-06	OTTMUST00000053605	1636	0	0	0	3				0.624			
Slc25a33	solute carrier family 25, member 33	-1.65	3.46E-06	0.000108	OTTMUST00000023896	702	0	0	0	0							
Pim3	proviral integration site 3	-1.65	2.09E-05	0.000354	ENSMUST00000042818	956	0	0	0	1				-0.55			
Lpin1	lipin 1	-1.65	5.31E-05	0.000656	ENSMUST00000048538	1777	0	0	0	1				-0.711			
Slamf9	SLAM family member 9	-1.65	0.000162	0.00146	OTTMUST00000050810	316	0	0	0	0							
Cenpv	centromere protein V	-1.64	3.80E-08	7.12E-06	OTTMUST00000013042	217	0	1	0	0						-0.302	
Dpy30	dpy-30 homolog (C. elegans)	-1.63	1.32E-07	1.43E-05	NM_024428	278	0	1	0	0						-0.31	
Crat	carnitine acetyltransferase	-1.63	2.71E-07	2.11E-05	OTTMUST00000040750	2107	0	0	0	1				-0.348			
Lrrc14b	leucine rich repeat containing 14B	-1.63	4.05E-07	2.67E-05	ENSMUST00000022064	1173	0	0	0	1							
Echs1	enoyl Coenzyme A hydratase, short chain, 1, mitochondrial	-1.63	5.32E-07	3.19E-05	OTTMUST00000067462	602	1	0	0	1	-2.147			-1.104			
Mrpl3	mitochondrial ribosomal protein L3	-1.63	7.08E-07	3.66E-05	OTTMUST00000057317	317	0	0	0	0							
Kcnip2	Kv channel-interacting protein 2	-1.63	1.29E-05	0.000254	ENSMUST00000026247	1324	0	0	0	0							
Cs	citrate synthase	-1.63	3.45E-05	0.000493	ENSMUST00000005826	1346	0	0	0	1				0.31			
Ccl11	chemokine (C-C motif) ligand 11	-1.63	6.48E-05	0.000749	OTTMUST00000001670	644	0	0	0	1							
Mgll	monoglyceride lipase	-1.62	6.26E-07	3.42E-05	OTTMUST00000058395	2584	0	0	0	2				1.42			
Bdnf	brain derived neurotrophic factor	-1.62	7.29E-06	0.00018	OTTMUST00000035871	2895	0	0	0	1				1.437			
Tbrg4	transforming growth factor beta regulated gene 4	-1.62	4.62E-05	0.000602	OTTMUST00000011462	190	0	0	0	0							
LOC381792		-1.62	4.93E-05	0.000628	XM_358653												
Acy1	aminoacylase 1	-1.61	1.82E-08	4.40E-06	ENSMUST00000024031	90	0	0	0	0							
Uqcrc1	ubiquinol-cytochrome c reductase core protein 1	-1.61	2.89E-07	2.13E-05	ENSMUST00000026743	122	0	0	0	0							
Ppargc1b	peroxisome proliferative activated receptor, gamma, coactivator 1 beta	-1.61	1.71E-06	6.44E-05	OTTMUST00000058693	547	0	0	0	0							
Rtn4ip1	reticulon 4 interacting protein 1	-1.61	5.77E-06	0.000154	OTTMUST00000055533	1334	0	0	0	0							
Tmem86a	transmembrane protein 86A	-1.6	1.17E-06	5.06E-05	ENSMUST00000010451	892	0	0	0	0							

Symbol	Description	FC	P.Value	adj.P.Val	Transcript	3'UTR_length	9mers	8mers	7mers	6mers	9mers_cons	8mers_cons	7mers_cons	6mers_cons	TS6_PCT	TSC_Context Plus_rerun	TargetScan_ORF
Ryr2	ryanodine receptor 2, cardiac	-1.6	1.42E-06	5.86E-05	ENSMUST00000021750	1413	0	0	0	1				0.72			
LOC227393		-1.6	3.92E-06	0.000118	XM_129965	261	0	0	0	0							
Lgals4	lectin, galactose binding, soluble 4	-1.6	3.19E-05	0.00047	OTTMUST00000054398	70	0	0	0	0							
Aldh1l2	aldehyde dehydrogenase 1 family, member L2	-1.6	0.000138	0.00129	OTTMUST00000061092	3129	0	1	0	1		-0.748				-0.197	
1700034H14Rik	RIKEN cDNA 1700034H14 gene	-1.59	9.77E-09	3.31E-06	ENSMUST00000025547	353	0	0	0	0							
Ndufa8	NADH dehydrogenase (ubiquinone) 1 alpha subcomplex, 8	-1.59	4.43E-07	2.79E-05	OTTMUST00000028784	178	0	0	0	0							
Tmem65	transmembrane protein 65	-1.59	6.00E-06	0.000156	OTTMUST00000049620	2640	0	0	0	1				-0.474			
Pdhb	pyruvate dehydrogenase (lipoamide) beta	-1.59	2.32E-05	0.000379	ENSMUST00000022268	338	0	0	0	0							
Pfkfb2	6-phosphofructo-2-kinase/fructose-2,6-biphosphatase 2	-1.59	0.000107	0.00108	ENSMUST00000066863	2835	0	0	0	1				0.048			
Mrpl47	mitochondrial ribosomal protein L47	-1.58	8.73E-06	0.000202	OTTMUST00000069652	2197	0	0	0	1				0.305			
Tmem143	transmembrane protein 143	-1.58	1.16E-05	0.000238	OTTMUST00000053174	782	0	0	0	2				0.616			
Camk2b	calcium/calmodulin-dependent protein kinase II, beta	-1.58	0.000101	0.00103	OTTMUST00000011419	2184	0	0	0	1				-0.319			
Tnks2	tankyrase, TRF1-interacting ankyrin-related ADP-ribose polymerase 2	-1.58	0.00019	0.00164	XM_917270	2696	0	1	0	1						-0.163	
Sbk1	SH3-binding kinase 1	-1.57	1.77E-08	4.38E-06	ENSMUST00000056028	2342	0	1	0	2		0.311		-0.278		-0.101	
Mrpl3	mitochondrial ribosomal protein L3	-1.57	1.95E-08	4.53E-06	OTTMUST00000057319	1242	0	0	0	1							
Ogdh	oxoglutarate dehydrogenase (lipoamide)	-1.57	0.00016	0.00144	OTTMUST00000001534	968	0	1	0	1		-1.118		-2.056		-0.169	
Pkm2	pyruvate kinase, muscle	-1.56	8.37E-09	3.31E-06	ENSMUST00000034834	538	0	0	0	2				0.686			
Idh1	isocitrate dehydrogenase 1 (NADP+), soluble	-1.56	1.65E-07	1.67E-05	OTTMUST00000052006	849	0	0	0	0							
Ank	progressive ankylosis	-1.56	1.99E-06	7.17E-05	OTTMUST00000065059	1692	0	0	0	1				0.369			
Tmod4	tropomodulin 4	-1.55	9.95E-05	0.00102	OTTMUST00000047077	99	0	0	0	0							
Slc4a3	solute carrier family 4 (anion exchanger), member 3	-1.54	5.25E-09	2.96E-06	OTTMUST00000046376	3282	0	0	0	0							
Prodh	proline dehydrogenase	-1.54	1.57E-06	6.10E-05	OTTMUST00000069796	450	0	0	0	1							
Fundc1	FUN14 domain containing 1	-1.54	4.52E-06	0.000127	OTTMUST00000041098	1370	0	0	1	1			-1.26			-0.189	
Gm766	predicted gene 766	-1.54	2.89E-05	0.000444	OTTMUST00000053389												
Cd74	CD74 antigen (invariant polypeptide of major histocompatibility complex, class II antigen-associated)	-1.54	0.000924	0.00528	ENSMUST00000097563	484	0	0	0	0							
Atp5j	ATP synthase, H+ transporting, mitochondrial F0 complex, subunit F	-1.53	1.11E-06	4.92E-05	OTTMUST00000062160	92	0	0	0	0							
A2bp1	ataxin 2 binding protein 1	-1.53	4.16E-06	0.000121	ENSMUST00000092375	274	0	0	0	0							
1300018J18Rik	RIKEN cDNA 1300018J18 gene	-1.53	4.28E-06	0.000122	OTTMUST00000049660	354	0	0	0	1				-1.035			
Rgma	RGM domain family, member A	-1.53	5.44E-06	0.000147	OTTMUST00000077776	1866	0	0	1	4				0.106		-0.058	
ENSMUSG00000068790	predicted gene, ENSMUSG00000068790	-1.53	1.29E-05	0.000254	ENSMUST00000112764	298	0	0	0	0							
Gpcpd1	glycerophosphocholine phosphodiesterase GDE1 homolog (S. cerevisiae)	-1.53	0.000183	0.00159	OTTMUST00000037199	1471	0	0	0	0							
Vegfa	vascular endothelial growth factor A	-1.53	0.000642	0.00398	OTTMUST00000039102	1865	0	0	0	0							
Afg3l1	AFG3(ATPase family gene 3)-like 1 (yeast)	-1.52	8.90E-07	4.28E-05	OTTMUST00000076944	1850	0	0	0	1							
Lrpprc	leucine-rich PPR-motif containing	-1.52	1.21E-06	5.21E-05	OTTMUST00000076447	169	0	0	0	0							
Adamts7	a disintegrin-like and metallopeptidase (reprolysin type) with thrombospondin type 1 motif, 7	-1.52	1.45E-06	5.88E-05	OTTMUST00000063704	231	0	0	0	0							
Pxmp2	peroxisomal membrane protein 2	-1.52	6.14E-06	0.000159	OTTMUST00000064832	278	0	0	0	1				-0.532			
Fyco1	FYVE and coiled-coil domain containing 1	-1.52	1.58E-05	0.000293	ENSMUST00000084715	3164	0	0	0	1				-0.942			
Ppfia1	protein tyrosine phosphatase, receptor type, f polypeptide (PTPRF), interacting protein (liprin), alpha 1	-1.52	3.02E-05	0.000457	ENSMUST00000093967	1198	0	0	0	1							
Gm3411	predicted gene 3411	-1.52	5.50E-05	0.000673	ENSMUST00000090647	746	0	0	0	0							
Retsat	retinol saturase (all trans retinol 13,14 reductase)	-1.52	0.000108	0.00108	OTTMUST00000066600	1033	0	0	0	0							
Atp5g1	ATP synthase, H+ transporting, mitochondrial F0 complex, subunit c (subunit 9), isoform 1	-1.51	1.01E-08	3.31E-06	OTTMUST00000014195	4252	0	0	1	1						-0.163	
Trnau1ap	tRNA selenocysteine 1 associated protein 1	-1.51	1.85E-06	6.78E-05	OTTMUST00000021958	107	0	0	0	0							
Mtg1	mitochondrial GTPase 1 homolog (S. cerevisiae)	-1.51	1.94E-06	7.04E-05	OTTMUST00000072052	513	0	0	0	1							
Ipo13	importin 13	-1.51	3.12E-06	1.00E-04	OTTMUST00000019617	75	0	0	0	0							
Gstk1	glutathione S-transferase kappa 1	-1.51	5.88E-06	0.000155	ENSMUST00000031897	162	0	0	0	0							
Supv3l1	suppressor of var1, 3-like 1 (S. cerevisiae)	-1.51	6.59E-06	0.000167	OTTMUST00000041801	211	0	0	0	0							
Abcb6	ATP-binding cassette, sub-family B (MDR/TAP), member 6	-1.51	8.17E-06	0.000193	ENSMUST00000027396	163	0	0	0	0							
4930534B04Rik	RIKEN cDNA 4930534B04 gene	-1.51	9.36E-06	0.000213	OTTMUST00000084463	1031	0	0	0	0							
Aplnr	apelin receptor	-1.51	1.93E-05	0.000336	OTTMUST00000032555	2157	0	1	0	2		0.237		-0.575		-0.252	
Dlst	dihydrolipoamide S-succinyltransferase (E2 component of 2-oxo-glutarate complex)	-1.51	5.58E-05	0.000678	ENSMUST00000053811	1330	0	0	0	0							
2610507B11Rik	RIKEN cDNA 2610507B11 gene	-1.51	0.000249	0.00199	OTTMUST00000000333	640	0	0	0	0							
Cdh4	cadherin 4	-1.51	0.000263	0.00207	OTTMUST00000038868	3455	0	2	0	2		-1.364		0.618		-0.179	
Rbm38	RNA binding motif protein 38	-1.51	0.00172	0.00838	OTTMUST00000040393	925	0	0	0	0							
Pigq	phosphatidylinositol glycan anchor biosynthesis, class Q	-1.5	1.06E-07	1.21E-05	OTTMUST00000082547	1176	0	1	0	0						-0.155	
Etfdh	electron transferring flavoprotein, dehydrogenase	-1.5	1.60E-07	1.66E-05	OTTMUST00000072852	214	0	0	0	0							
L2hgdh	L-2-hydroxyglutarate dehydrogenase	-1.5	3.86E-07	2.58E-05	NM_145443	1865	0	0	0	1							
P2ry14	purinergic receptor P2Y, G-protein coupled, 14	-1.5	1.43E-06	5.88E-05	ENSMUST00000065220	395	0	0	0	0							

Symbol	Description	FC	P.Value	adj.P.Val	Transcript	3'UTR_length	9mers	8mers	7mers	6mers	9mers_cons	8mers_cons	7mers_cons	6mers_cons	TS6_PCT	TSC_Context Plus_rerun	TargetScan_ ORF
Flcn	folliculin	-1.5	1.66E-06	6.40E-05	OTTMUST00000018400	1202	0	0	0	0							
Brp44	brain protein 44	-1.5	2.81E-06	9.25E-05	OTTMUST00000054065	275	0	0	0	0							
1110038D17Rik	RIKEN cDNA 1110038D17 gene	-1.5	3.19E-06	0.000101	OTTMUST00000078804	2595	0	1	1	0						-0.142	
Cox15	COX15 homolog, cytochrome c oxidase assembly protein (yeast)	-1.5	1.75E-05	0.000318	OTTMUST00000065269	3467	0	0	2	2			-0.24	0.145		-0.193	
Rtn2	reticulon 2 (Z-band associated protein)	-1.5	2.19E-05	0.000365	ENSMUST00000032559	358	0	0	0	0							
Spryd4	SPRY domain containing 4	-1.5	3.27E-05	0.000475	ENSMUST00000061995	939	0	0	1	0			0.288			-0.12	
Homer1	homer homolog 1 (Drosophila)	-1.5	0.000112	0.00111	OTTMUST00000039265	4349	0	2	0	2		0.424		-0.916		-0.366	
Casz1	castor homolog 1, zinc finger (Drosophila)	-1.5	0.000371	0.00265	OTTMUST00000025638	2318	0	0	1	0			0.911			-0.101	
Kcnk3	potassium channel, subfamily K, member 3	-1.5	0.000446	0.00304	ENSMUST00000066295	2433	0	1	0	2		-1.06		0.037		-0.101	
Lama5	laminin, alpha 5	-1.5	0.00167	0.00821	OTTMUST00000039036	174	0	0	0	0							

Only those genes dysregulated 1.5-fold or greater are listed in table.
FC: Fold change over WT; P value: raw p-value; adj.Pvalue: p-value adjusted to false discovery rate
9mers, 8mers, 7mers, 6mers: the number of miR-22 seed-matching sites in 3'UTR, color is according to their size (red, 6mers; blue, 7mers; green, 8mers; orange, 9mers.
9/8/7/6mers_cons: Average GERP conservation scores across miR-22 seed matching sites, according to genomic alignment. If the 3'UTR has more than one site, highest score is reported.
TS6_PCT: Probability of Conserved Targeting, according to TargetScan 6.0
TS6_ContextPlus: Context+ scores from TargetScan 6.0
TS6_ContextPlus_rerun: To account for differences between our sequences with those used by TargetScan, we re-ran the targetscan_60_context_scores.pl script to obtain these Context+ scores for exactly our 3'UTR sequences.
TargetScan_ORF: Genes reported to have a conserved seed target in their coding region, see Methods
Microarray data was deposited in Array Express: E-MTAB-501

Table S2. Genes disregulated in *miR-22*-null hearts (continued)

Up-regulated genes (genes with greater 2-fold induction are shown)

Symbol	Description	FC	P.Value	adj.P.Val	Transcript
Myh7	myosin, heavy polypeptide 7, cardiac muscle, beta	17.2	8.41E-09	3.31E-06	OTTMUST00000039449
Nppa	natriuretic peptide precursor type A	8.71	2.35E-06	8.13E-05	OTTMUST00000023685
Scgb1c1	secretoglobin, family 1C, member 1	7.75	1.94E-10	9.69E-07	ENSMUST00000035300
Rcan1	regulator of calcineurin 1	7.44	6.45E-08	9.55E-06	NM_019466
Nmral1	NmrA-like family domain containing 1	5.16	2.60E-10	9.69E-07	OTTMUST00000065955
2610028H24Rik	RIKEN cDNA 2610028H24 gene	5.13	0.000491	0.00326	OTTMUST00000048272
Acta1	actin, alpha 1, skeletal muscle	5.04	2.69E-09	2.74E-06	ENSMUST00000034453
Nppb	natriuretic peptide precursor type B	4.25	1.87E-09	2.26E-06	OTTMUST00000023693
Ctgf	connective tissue growth factor	4.24	3.12E-06	1.00E-04	OTTMUST00000047191
Acta2	actin, alpha 2, smooth muscle, aorta	4.19	9.11E-09	3.31E-06	NM_007392
Acta2	actin, alpha 2, smooth muscle, aorta	3.99	1.76E-09	2.26E-06	ENSMUST00000039631
Itgb1bp3	integrin beta 1 binding protein 3	3.95	6.13E-10	1.12E-06	ENSMUST00000005069
Fhl1	four and a half LIM domains 1	3.93	2.54E-06	8.64E-05	OTTMUST00000042746
Slc24a2	solute carrier family 24 (sodium/potassium/calcium exchanger), member 2	3.4	5.62E-08	8.66E-06	OTTMUST00000017530
Rcan1	regulator of calcineurin 1	3.16	9.42E-08	1.16E-05	ENSMUST00000060005
Spsb4	splA/ryanodine receptor domain and SOCS box containing 4	3.15	2.83E-06	9.29E-05	OTTMUST00000071893
Lman1l	lectin, mannose-binding 1 like	2.95	7.04E-07	3.66E-05	OTTMUST00000074318
Pdlim3	PDZ and LIM domain 3	2.83	9.29E-09	3.31E-06	ENSMUST00000034053
Cp	ceruloplasmin	2.71	2.28E-07	1.88E-05	OTTMUST00000057275
Cnksr1	connector enhancer of kinase suppressor of Ras 1	2.67	9.99E-09	3.31E-06	OTTMUST00000024609
Xirp2	xin actin-binding repeat containing 2	2.67	9.91E-07	4.54E-05	OTTMUST00000030942
Sypl2	synaptophysin-like 2	2.6	7.49E-08	9.96E-06	OTTMUST00000016565
Arhgap24	Rho GTPase activating protein 24	2.59	9.49E-09	3.31E-06	OTTMUST00000055707
1500017E21Rik	RIKEN cDNA 1500017E21 gene	2.57	6.15E-10	1.12E-06	OTTMUST00000070864
Myot	myotilin	2.45	5.69E-09	2.96E-06	OTTMUST00000035442
Etv5	ets variant gene 5	2.45	8.88E-06	0.000204	ENSMUST00000051229
C4b	complement component 4B (Childo blood group)	2.45	0.00429	0.0168	ENSMUST00000069507
LOC626152	similar to Epiplakin	2.4	1.45E-06	5.88E-05	XR_032670
Sort1	sortilin 1	2.34	1.78E-07	1.75E-05	OTTMUST00000016875
Mybpc2	myosin binding protein C, fast-type	2.33	6.02E-07	3.36E-05	NM_178067
Dpysl3	dihydropyrimidinase-like 3	2.28	1.89E-05	0.000333	OTTMUST00000073510
Fam122b	family with sequence similarity 122, member B	2.27	5.19E-05	0.000647	OTTMUST00000042133
Naca	nascent polypeptide-associated complex alpha polypeptide	2.25	2.33E-06	8.09E-05	NM_013608
Gnmt	glycine N-methyltransferase	2.23	5.92E-05	0.000706	OTTMUST00000065249
1500009L16Rik	RIKEN cDNA 1500009L16 gene	2.22	3.88E-09	2.78E-06	XM_899407
Hn1	hematological and neurological expressed sequence 1	2.19	1.52E-06	6.02E-05	OTTMUST00000007275
Polr1e	polymerase (RNA) I polypeptide E	2.18	9.50E-05	0.000986	OTTMUST00000017087
C4a	complement component 4A (Rodgers blood group)	2.18	0.0129	0.0395	ENSMUST00000015595
Atpif1	ATPase inhibitory factor 1	2.17	2.67E-10	9.69E-07	OTTMUST00000022663
Pdlim1	PDZ and LIM domain 1 (elfin)	2.17	1.82E-06	6.74E-05	ENSMUST00000068439

Symbol	Description	FC	P.Value	adj.P.Val	Transcript
Tmem176b	transmembrane protein 176B	2.13	4.46E-07	2.80E-05	ENSMUST00000031833
Tspan17	tetraspanin 17	2.12	6.81E-05	0.000777	OTTMUST00000081270
Serpina3n	serine (or cysteine) peptidase inhibitor, clade A, member 3N	2.12	0.00432	0.0169	OTTMUST00000039207
Itgb5	integrin beta 5	2.11	7.14E-06	0.000178	OTTMUST00000070600
Rrp12	ribosomal RNA processing 12 homolog (S. cerevisiae)	2.1	1.01E-05	0.000223	ENSMUST00000038677
Dnaja4	DnaJ (Hsp40) homolog, subfamily A, member 4	2.08	4.45E-06	0.000126	OTTMUST00000067744
Slc1a1	solute carrier family 1 (neuronal/epithelial high affinity glutamate transporter, system Xag), member 1	2.08	3.24E-05	0.000474	OTTMUST00000085484
Nuak1	NUAK family, SNF1-like kinase, 1	2.07	4.20E-06	0.000122	OTTMUST00000054650
Pbxip1	pre-B-cell leukemia transcription factor interacting protein 1	2.07	7.39E-06	0.000181	OTTMUST00000052415
Ccl21a	chemokine (C-C motif) ligand 21A	2.07	0.000966	0.00545	OTTMUST00000015039
Prkcz	protein kinase C, zeta	2.06	3.26E-07	2.31E-05	OTTMUST00000024748
H19	H19 fetal liver mRNA	2.06	4.39E-06	0.000125	OTTMUST00000035613
Lrp11	low density lipoprotein receptor-related protein 11	2.04	9.92E-05	0.00102	OTTMUST00000081563
Maged2	melanoma antigen, family D, 2	2.03	2.63E-06	8.85E-05	OTTMUST00000086091
Ppfibp1	PTPRF interacting protein, binding protein 1 (liprin beta 1)	2.02	1.81E-07	1.76E-05	OTTMUST00000055355
Adamtsl5	ADAMTS-like 5	2.01	5.06E-08	8.36E-06	OTTMUST00000045831

Only those genes dysregulated 2.0-fold or greater are listed in table.

FC: Fold change over WT; P value: raw p-value; adj.Pvalue: p-value adjusted to false discovery rate
