# Supplementary material for: microRNA-22 Promotes Heart Failure through Coordinate Suppression of PPAR/ERR-Nuclear Hormone Receptor Transcription
Source: PLoS One. 2013 Sep 27;8(9):e75882. doi: 10.1371/journal.pone.0075882 (PMC3785418; doi:10.1371/journal.pone.0075882)
Supplement: Table S3 — Potential Targets of miR-22 in the heart. (PDF) [file pone.0075882.s008.pdf]

Table S3. Potential targets of *miR-22* in the heart

Entrez Gene Name	Location	Family	Cellular Growth and Proliferation	Cell Death	Lipid Metabolism	Molecule Biochemistry	Cardiovascular Disease
abhydrolase domain containing 8	unknown	enzyme					
acyl-Coenzyme A dehydrogenase family, member 8	Cytoplasm	enzyme					
acyl-Coenzyme A dehydrogenase family, member 9	Cytoplasm	enzyme					
acyl-Coenzyme A dehydrogenase, long chain	Cytoplasm	enzyme					
acyl-CoA synthetase long-chain family member 6	Cytoplasm	enzyme					
alcohol dehydrogenase, iron containing, 1	unknown	enzyme					
adenosine kinase	Nucleus	kinase					
AHNAK nucleoprotein	Nucleus	other					
aryl-hydrocarbon receptor repressor	Nucleus	other					
apoptosis-inducing factor, mitochondrion-associated, 2	Cytoplasm	enzyme					
aldehyde dehydrogenase 1 family, member L2	unknown	enzyme					
aldehyde dehydrogenase 2 family (mitochondrial)	Cytoplasm	enzyme					
aldehyde dehydrogenase 5 family, member A1	Cytoplasm	enzyme					
alkB, alkylation repair homolog 5 (E. coli)	unknown	other					
arachidonate 5-lipoxygenase	Cytoplasm	enzyme					
anaphase promoting complex subunit 5	Nucleus	enzyme					
ankyrin repeat domain 50	unknown	other					
amyloid beta (A4) precursor protein-binding, family B, member 2	Cytoplasm	other					
apelin receptor	Plasma Membrane	G-protein coupled receptor					
ArfGAP with RhoGAP domain, ankyrin repeat and PH domain 3	Nucleus	other					
activity-regulated cytoskeleton-associated protein	Cytoplasm	other					
ADP-ribosylation factor GTPase activating protein 2	Nucleus	other					
ADP-ribosylation factor related protein 1	Cytoplasm	enzyme					
Rho guanine nucleotide exchange factor (GEF) 15	Cytoplasm	other					
ankyrin repeat and SOCS box-containing 13	unknown	other					
ankyrin repeat and SOCS box-containing 8	Nucleus	transcription regulator					
ATPase, Na+/K+ transporting, alpha 2 (+) polypeptide	Plasma Membrane	transporter					
ATPase, Ca++ transporting, ubiquitous	Cytoplasm	transporter					
ATP synthase, H+ transporting, mitochondrial F0 complex, subunit C1 (subunit 9)	Cytoplasm	transporter					
ATPase, class II, type 9B	Cytoplasm	transporter					
ataxin 1	Nucleus	other					
apoptosis, caspase activation inhibitor	Cytoplasm	ion channel					
BTB and CNC homology 1, basic leucine zipper transcription factor 1	Nucleus	transcription regulator					
BRCA1 associated protein-1 (ubiquitin carboxy-terminal hydrolase)	Nucleus	peptidase					
BCL2-like 11 (apoptosis facilitator)	Cytoplasm	other					
B-cell CLL/lymphoma 9-like	Cytoplasm	other					
bromodomain containing 4	Nucleus	kinase					
calmodulin 3 (phosphorylase kinase, delta)	Plasma Membrane	other					
calmodulin binding transcription activator 2	unknown	other					
CASK interacting protein 2	Cytoplasm	other					
castor zinc finger 1	Nucleus	enzyme					
caveolin 3	Plasma Membrane	enzyme					
cyclin G1	Nucleus	other					
CD82 molecule	Plasma Membrane	other					
CD93 molecule	Plasma Membrane	other					
cadherin 4, type 1, R-cadherin (retinal)	Plasma Membrane	other					
CDKN2A interacting protein N-terminal like	unknown	other					
centromere protein A	Nucleus	other					
centromere protein V	Nucleus	other					
chromodomain helicase DNA binding protein 7	Nucleus	enzyme					
chromatin accessibility complex 1	Nucleus	enzyme					
cytokine induced apoptosis inhibitor 1	Cytoplasm	other					
claudin domain containing 1	Plasma Membrane	other					
C-type lectin domain family 16, member A	unknown	other					
chloride intracellular channel 4	Cytoplasm	ion channel					
contactin 2 (axonal)	Plasma Membrane	other					
coronin 6	unknown	other					
COX15 homolog, cytochrome c oxidase assembly protein (yeast)	Cytoplasm	enzyme					
cytoplasmic polyadenylation element binding protein 3	unknown	other					
calcineurin-like phosphoesterase domain containing 1	unknown	enzyme					
cold shock domain containing C2, RNA binding	Cytoplasm	other					
colony stimulating factor 1 (macrophage)	Extracellular Space	cytokine					
cystinosis, nephropathic	Cytoplasm	transporter					
cut-like homeobox 1	Nucleus	transcription regulator					
chemokine (C-X-C motif) ligand 12 (stromal cell-derived factor 1)	Extracellular Space	cytokine					
cytochrome P450, family 2, subfamily S, polypeptide 1	Cytoplasm	enzyme					
D-aspartate oxidase	Cytoplasm	enzyme					
DEAD (Asp-Glu-Ala-Asp) box polypeptide 49	Nucleus	enzyme					
DENN/MADD domain containing 3	unknown	other					
DEAH (Asp-Glu-Ala-His) box polypeptide 35	unknown	enzyme					
DnaJ (Hsp40) homolog, subfamily A, member 3	Cytoplasm	other					
DnaJ (Hsp40) homolog, subfamily C, member 27	unknown	enzyme					
DnaJ (Hsp40) homolog, subfamily C, member 28	unknown	other					
DnaJ (Hsp40) homolog, subfamily C, member 7	Cytoplasm	other					
dolichyl-phosphate mannosyltransferase polypeptide 2, regulatory subunit	Cytoplasm	enzyme					
dpy-30 homolog (C. elegans)	Nucleus	other					
dihydropyrimidinase-like 4	Cytoplasm	enzyme					
dual specificity phosphatase 28	unknown	other					
enoyl Coenzyme A hydratase, short chain, 1, mitochondrial	Cytoplasm	enzyme					
enhancer of mRNA decapping 3 homolog (S. cerevisiae)	Cytoplasm	other					
EF-hand calcium binding domain 2	unknown	other					
ephrin-A1	Plasma Membrane	other					
epidermal growth factor receptor pathway substrate 15-like 1	Plasma Membrane	other					
excision repair cross-complementing rodent repair deficiency, complementation group 8	Nucleus	transcription regulator					
ER lipid raft associated 2	Plasma Membrane	other					
electron-transfer-flavoprotein, alpha polypeptide	Cytoplasm	transporter					
v-ets erythroblastosis virus E26 oncogene homolog 1 (avian)	Nucleus	transcription regulator					
family with sequence similarity 101, member B	unknown	other					
family with sequence similarity 168, member B	unknown	other					
family with sequence similarity 49, member B	unknown	other					
family with sequence similarity 53, member C	unknown	other					
F-box and WD repeat domain containing 5	Cytoplasm	other					
fms-related tyrosine kinase 1 (vascular endothelial growth factor/vascular permeability factor receptor)	Plasma Membrane	kinase					

Entrez Gene Name	Location	Family	Cellular Growth and Proliferation	Cell Death	Lipid Metabolism	Molecule Biochemistry	Cardiovascular Disease
fibronectin type III domain containing 5	unknown	other					
FAD-dependent oxidoreductase domain containing 1	Cytoplasm	other					
FUN14 domain containing 1	unknown	other					
furin (paired basic amino acid cleaving enzyme)	Cytoplasm	peptidase					
galactose mutarotase (aldose 1-epimerase)	Cytoplasm	enzyme					
glutamyl-tRNA(Gln) amidotransferase, subunit C homolog (bacterial)	Nucleus	other					
glioblastoma amplified sequence	Plasma Membrane	other					
glucokinase (hexokinase 4)	Cytoplasm	kinase					
glycerophosphodiester phosphodiesterase domain containing 1	unknown	enzyme					
G elongation factor, mitochondrial 2	Cytoplasm	translation regulator					
glutaredoxin (thioltransferase)	Cytoplasm	enzyme					
glutaredoxin 2	Cytoplasm	enzyme					
gon-4-like (C. elegans)	unknown	other					
GC-rich promoter binding protein 1	Nucleus	transcription regulator					
glypican 1	Plasma Membrane	transmembrane receptor					
G protein-coupled receptor 22	Plasma Membrane	G-protein coupled receptor					
G-protein signaling modulator 1 (AGS3-like, C. elegans)	Cytoplasm	other					
GSG1-like	unknown	other					
GTP binding protein 1	Cytoplasm	enzyme					
H3 histone, family 3B (H3.3B)	Nucleus	other					
hairy/enhancer-of-split related with YRPW motif 1	Nucleus	transcription regulator					
3-hydroxyisobutyrate dehydrogenase	Cytoplasm	enzyme					
H2.0-like homeobox	Nucleus	transcription regulator					
heterogeneous nuclear ribonucleoprotein A3	Nucleus	other					
homer homolog 1 (Drosophila)	Plasma Membrane	other					
HRAS-like suppressor	Cytoplasm	other					
heparan sulfate proteoglycan 2	Plasma Membrane	other					
HIV-1 Tat interactive protein 2, 30kDa	Nucleus	transcription regulator					
HUS1 checkpoint homolog (S. pombe)	Nucleus	kinase					
intraflagellar transport 20 homolog (Chlamydomonas)	Cytoplasm	other					
integrin alpha FG-GAP repeat containing 2	unknown	other					
integrin, beta 6	Plasma Membrane	other					
inositol 1,4,5-trisphosphate 3-kinase B	Cytoplasm	kinase					
inositol 1,4,5-triphosphate receptor, type 2	Cytoplasm	ion channel					
inositol 1,4,5-triphosphate receptor interacting protein-like 1	unknown	other					
junctional adhesion molecule 2	Plasma Membrane	other					
potassium inwardly-rectifying channel, subfamily J, member 8	Plasma Membrane	ion channel					
potassium channel, subfamily K, member 3	Plasma Membrane	ion channel					
potassium channel, subfamily V, member 2	Cytoplasm	ion channel					
potassium channel tetramerisation domain containing 10	unknown	ion channel					
lysine (K)-specific demethylase 3A	unknown	other					
lysine (K)-specific demethylase 6B	unknown	other					
kelch domain containing 10	unknown	other					
kelch domain containing 3	Cytoplasm	other					
KRAB-A domain containing 1	unknown	other					
limb bud and heart development homolog (mouse)	Nucleus	transcription regulator					
Ly6/neurotoxin 1	Plasma Membrane	transporter					
mitogen-activated protein kinase kinase kinase 1	Cytoplasm	kinase					
MARVEL domain containing 1	unknown	other					
MCF.2 cell line derived transforming sequence-like	Cytoplasm	other					
Meis homeobox 2	Nucleus	transcription regulator					
microfibrillar-associated protein 3-like	unknown	other					
major facilitator superfamily domain containing 11	unknown	other					
major facilitator superfamily domain containing 7C							
monoglyceride lipase	Plasma Membrane	enzyme					
matrix metallopeptidase 15 (membrane-inserted)	Extracellular Space	peptidase					
melanoregulin	unknown	other					
mitochondrial rRNA methyltransferase 1 homolog (S. cerevisiae)	unknown	other					
mitochondrial ribosomal protein S26	Cytoplasm	other					
mitochondrial ribosome recycling factor	Cytoplasm	other					
mitochondrial carrier homolog 2 (C. elegans)	Cytoplasm	other					
metallothionein-like 5, testis-specific (tesmin)	Cytoplasm	other					
myosin XVIIIA	Cytoplasm	other					
N(alpha)-acetyltransferase 10, NatA catalytic subunit	Nucleus	enzyme					
N(alpha)-acetyltransferase 20, NatB catalytic subunit	Cytoplasm	enzyme					
neural cell adhesion molecule 1	Plasma Membrane	other					
nuclear receptor coactivator 1	Nucleus	transcription regulator					
nudE nuclear distribution gene E homolog 1 (A. nidulans)	Nucleus	other					
NADH dehydrogenase (ubiquinone) 1, alpha/beta subcomplex, 1, 8kDa	Cytoplasm	enzyme					
NADH dehydrogenase (ubiquinone) Fe-S protein 5, 15kDa (NADH-coenzyme Q reductase)	Cytoplasm	enzyme					
NECAP endocytosis associated 1	Plasma Membrane	other					
neural precursor cell expressed, developmentally down-regulated 1	Cytoplasm	other					
nuclear transcription factor Y, beta	Nucleus	transcription regulator					
Notch homolog 4 (Drosophila)	Plasma Membrane	transcription regulator					
Niemann-Pick disease, type C1	Cytoplasm	transporter					
nudix (nucleoside diphosphate linked moiety X)-type motif 16-like 1	Cytoplasm	other					
nudix (nucleoside diphosphate linked moiety X)-type motif 4	Cytoplasm	phosphatase					
nuclear mitotic apparatus protein 1	Nucleus	other					
oxoglutarate (alpha-ketoglutarate) dehydrogenase (lipoamide)	Cytoplasm	enzyme					
osteoglycin	Extracellular Space	growth factor					
optineurin	Cytoplasm	other					
oxysterol binding protein-like 3	Cytoplasm	other					
OTU domain containing 4	unknown	other					
PAN2 poly(A) specific ribonuclease subunit homolog (S. cerevisiae)	Cytoplasm	peptidase					
protocadherin 12	Plasma Membrane	other					
progressive external ophthalmoplegia 1 (human)	unknown	other					
pyruvate dehydrogenase (lipoamide) alpha 1	Cytoplasm	enzyme					
pyruvate dehydrogenase kinase, isozyme 1	Cytoplasm	kinase					
pyruvate dehyrogenase phosphatase catalytic subunit 2	Cytoplasm	phosphatase					
prenyl (decaprenyl) diphosphate synthase, subunit 1	unknown	enzyme					
PET112-like (yeast)	Cytoplasm	translation regulator					
peroxisomal biogenesis factor 6	Cytoplasm	enzyme					
placental growth factor	Extracellular Space	growth factor					
phosphatidylinositol glycan anchor biosynthesis, class Q	Cytoplasm	enzyme					
phosphoinositide-3-kinase, regulatory subunit 1 (alpha)	Cytoplasm	kinase					
phosphatidylinositol transfer protein, cytoplasmic 1	Cytoplasm	transporter					
pyruvate kinase, muscle	Cytoplasm	kinase					
phospholipase A2, group V	Extracellular Space	enzyme					
phospholipase C, beta 3 (phosphatidylinositol-specific)	Cytoplasm	enzyme					
pleckstrin homology domain containing, family A (phosphoinositide binding specific) member 8	unknown	other					
pleckstrin homology domain containing, family H (with MyTH4 domain) member 1	Cytoplasm	other					
perilipin 2	Plasma Membrane	other					

Entrez Gene Name	Location	Family	Cellular Growth and Proliferation	Cell Death	Lipid Metabolism	Molecule Biochemistry	Cardiovascular Disease
plexin A2	Plasma Membrane	other					
phosphomannomutase 2	Cytoplasm	enzyme					
paroxysmal nonkinesigenic dyskinesia	Nucleus	other					
pogo transposable element with KRAB domain	Nucleus	other					
peroxisome proliferator-activated receptor alpha	Nucleus	transcription regulator					
peroxisome proliferator-activated receptor gamma, coactivator 1 alpha	Nucleus	transcription regulator					
protein phosphatase, Mg2+/Mn2+ dependent, 1K	unknown	phosphatase					
protein phosphatase 1, regulatory (inhibitor) subunit 16B	Plasma Membrane	phosphatase					
protein kinase D2	Cytoplasm	kinase					
protein-kinase, interferon-inducible double stranded RNA dependent inhibitor, repressor of (P58 repressor)	Nucleus	other					
prolactin receptor	Plasma Membrane	transmembrane receptor					
protein arginine methyltransferase 7	Cytoplasm	enzyme					
PRP19/PSO4 pre-mRNA processing factor 19 homolog (S. cerevisiae)	Nucleus	other					
PRP38 pre-mRNA processing factor 38 (yeast) domain containing A	Nucleus	other					
pentatricopeptide repeat domain 2	unknown	other					
protein tyrosine phosphatase, non-receptor type 11	Cytoplasm	phosphatase					
purine-rich element binding protein B	Nucleus	transcription regulator					
RAB3D, member RAS oncogene family	Cytoplasm	enzyme					
RAP1 GTPase activating protein	Cytoplasm	other					
Rap guanine nucleotide exchange factor (GEF) 3	Nucleus	other					
RASD family, member 2	Cytoplasm	enzyme					
RAS guanyl releasing protein 3 (calcium and DAG-regulated)	Cytoplasm	other					
Ras association (RalGDS/AF-6) domain family (N-terminal) member 8	unknown	other					
RNA binding motif protein 18	unknown	other					
receptor accessory protein 1	Cytoplasm	other					
RGM domain family, member A	Plasma Membrane	other					
regulator of G-protein signaling 5	Plasma Membrane	other					
regulator of G-protein signaling 7 binding protein	unknown	other					
rhomboid, veinlet-like 3 (Drosophila)	Plasma Membrane	peptidase					
ras homolog gene family, member B	Cytoplasm	enzyme					
resistance to inhibitors of cholinesterase 8 homolog B (C. elegans)	unknown	other					
ring finger protein 187	unknown	other					
RNA polymerase II associated protein 1	unknown	other					
RNA pseudouridylate synthase domain containing 4	unknown	other					
ribonucleotide reductase M2 B (TP53 inducible)	Nucleus	enzyme					
radical S-adenosyl methionine domain containing 2	unknown	enzyme					
reticulon 3	Cytoplasm	other					
SH3-binding domain kinase 1	unknown	kinase					
secretory carrier membrane protein 1	Cytoplasm	transporter					
sodium channel, voltage-gated, type IV, beta	Plasma Membrane	ion channel					
sodium channel, voltage-gated, type V, alpha subunit	Plasma Membrane	ion channel					
SEC24 family, member C (S. cerevisiae)	Cytoplasm	transporter					
sema domain, immunoglobulin domain (Ig), transmembrane domain (TM) and short cytoplasmic domain, (semaphorin) 4A	Plasma Membrane	other					
secreted frizzled-related protein 1	Plasma Membrane	transmembrane receptor					
small glutamine-rich tetratricopeptide repeat (TPR)-containing, alpha	Cytoplasm	other					
SH3-binding domain protein 5-like	unknown	other					
SH3-domain kinase binding protein 1	Cytoplasm	other					
SH3 and multiple ankyrin repeat domains 3	Cytoplasm	transcription regulator					
sirtuin 1 (silent information regulation 2, homolog 1)	Nucleus	transcription regulator					
solute carrier family 12 (potassium/chloride transporters), member 7	Plasma Membrane	transporter					
solute carrier family 26, member 10	unknown	transporter					
solute carrier family 35 (UDP-N-acetylglucosamine (UDP-GlcNAc) transporter), member A3	Cytoplasm	transporter					
solute carrier family 44, member 1	Plasma Membrane	transporter					
solute carrier family 8 (sodium/calcium exchanger), member 1	Plasma Membrane	transporter					
snail homolog 3 (Drosophila)	Nucleus	transcription regulator					
sorbin and SH3 domain containing 1	Plasma Membrane	other					
spectrin beta 1	unknown	other					
SPRY domain containing 4	unknown	other					
serum response factor (c-fos serum response element-binding transcription factor)	Nucleus	transcription regulator					
sulfiredoxin 1 homolog (S. cerevisiae)	Cytoplasm	enzyme					
signal sequence receptor, beta (translocon-associated protein beta)	Cytoplasm	other					
ST3 beta-galactoside alpha-2,3-sialyltransferase 5	Cytoplasm	enzyme					
starch binding domain 1	Cytoplasm	other					
serine threonine kinase 39 (STE20/SPS1 homolog, yeast)	Nucleus	kinase					
TGF-beta activated kinase 1/MAP3K7 binding protein 1	Cytoplasm	enzyme					
tachykinin, precursor 1	Extracellular Space	other					
tudor and KH domain containing	unknown	other					
thyrotrophic embryonic factor	Nucleus	transcription regulator					
transcription factor Dp-1	Nucleus	transcription regulator					
translocase of inner mitochondrial membrane 44 homolog (yeast)	Cytoplasm	transporter					
transmembrane protein 101	Extracellular Space	other					
transmembrane protein 143	Cytoplasm	other					
transmembrane protein 144	unknown	other					
transmembrane protein 199	unknown	other					
transmembrane protein 201	unknown	other					
transmembrane protein 50B	Plasma Membrane	other					
transmembrane protein 64	unknown	other					
TMEM9 domain family, member B	Plasma Membrane	other					
TRAF2 and NCK interacting kinase	Cytoplasm	kinase					
tankyrase, TRF1-interacting ankyrin-related ADP-ribose polymerase 2	Nucleus	enzyme					
translocase of outer mitochondrial membrane 6 homolog (yeast)	unknown	other					
tumor protein p53 inducible nuclear protein 1	Nucleus	other					
tribbles homolog 2 (Drosophila)	Plasma Membrane	kinase					
uracil-DNA glycosylase	Nucleus	enzyme					
vesicle transport through interaction with t-SNAREs homolog 1A (yeast)	Plasma Membrane	transporter					
wingless-type MMTV integration site family, member 5A	Extracellular Space	other					
exportin 7	Nucleus	transporter					
zinc finger, CCHC domain containing 8	unknown	other					
zinc finger protein 131	unknown	other					

Genes with only one 6-mer sequence in the 3'UTR were excluded from this list.
